# Exploring selective autophagy events in multiple biologic models using LC3-interacting regions (LIR)-based molecular traps

**DOI:** 10.1038/s41598-022-11417-z

**Published:** 2022-05-10

**Authors:** Grégoire Quinet, Pierre Génin, Oznur Ozturk, Naima Belgareh-Touzé, Lilas Courtot, Renaud Legouis, Robert Weil, Mickael M. Cohen, Manuel S. Rodriguez

**Affiliations:** 1 Laboratoire de Chimie de Coordination (LCC)-CNRS, UPS, 31400 Toulouse, France; 2 Centre d’immunologie et des Maladies Infectieuses (CIMI-Paris)- Faculté de Médecine, Sorbonne Université, 75013 Paris, France; 3 Sorbonne Université, CNRS, UMR8226, Institut de Biologie Physico-Chimique, Laboratoire de Biologie Moléculaire et Cellulaire des Eucaryotes, 75005 Paris, France; 4 Institute for Integrative Biology of the Cell (I2BC), Université Paris-Saclay, CEA, CNRS, 91198 Gif-sur-Yvette, France; 5 INSERM U1280, 91198 Gif‐sur‐Yvette, France

**Keywords:** Biochemistry, Proteases, Autophagy, Macroautophagy

## Abstract

Autophagy is an essential cellular pathway that ensures degradation of a wide range of substrates including damaged organelles or large protein aggregates. Understanding how this proteolytic pathway is regulated would increase our comprehension on its role in cellular physiology and contribute to identify biomarkers or potential drug targets to develop more specific treatments for disease in which autophagy is dysregulated. Here, we report the development of molecular traps based in the tandem disposition of LC3-interacting regions (LIR). The estimated affinity of LC3-traps for distinct recombinant LC3/GABARAP proteins is in the low nanomolar range and allows the capture of these proteins from distinct mammalian cell lines*, S. cerevisiae* and *C. elegans*. LC3-traps show preferences for GABARAP/LGG1 or LC3/LGG2 and pull-down substrates targeted to proteaphagy and mitophagy. Therefore, LC3-traps are versatile tools that can be adapted to multiple applications to monitor selective autophagy events in distinct physiologic and pathologic circumstances.

## Introduction

Protein homeostasis or ‘proteostasis’ maintains an equilibrium of the proteome and regulate multiple processes within the cell. Protein degradation plays a crucial role in the regulation of this equilibrium that is heavily regulated by distinct post-translational modifications including ubiquitin and various ubiquitin-like proteins (UbLs)^1,2^. Protein ubiquitylation was initially associated to proteasomal degradation, but over the last years, accumulated evidence underlined its importance in autophagy-mediated degradation. Autophagy drives the degradation of large cargoes, including organelles, molecular complexes or protein aggregates^3^. A basal level of autophagy occurs in resting cells, which is enhanced by stress conditions such as starvation, infections, heat shock or drug treatment. Dysregulation of autophagy is implicated in a number of pathologies, including inflammatory diseases, cancer, multiple infections, or neurodegenerative disorders. Autophagy is a conserved process that exists in mammalians, plants, insects and yeast. Early studies supported the notion that autophagy was a non-selective pathway, in which cargoes were randomly degraded. However, multiple evidences demonstrate an intricate selectivity in many cases, where specific structures are specifically recognized and eliminated by the autophagy-lysosome pathway. Hence, selective autophagy can be categorized in several types, such as mitophagy, xenophagy, aggregaphagy, proteaphagy, among others, depending on their specific cargo^4^. Some of these events have been associated to pathologies, for instance, mitophagy and neurodegenerative diseases including Amyotrophic Lateral Sclerosis and Parkinson disease^5^.

Autophagy-related proteins (ATG) ensure the formation of a large cup-shaped double membrane named phagophore. Cargoes recognition precedes autophagosome maturation. Substrates are sequestered in autophagosomes, and degraded after fusion with either endosomes and/or lysosomes/vacuoles where substrate degradation occurs^6^. Among the ATG proteins, the UbLs ATG8 proteins (LC3/GABARAP in mammalians) are the most used to monitor autophagy, through their post-translational modifications. Composed of 6 members, LC3A, B and C, and GABARAP, GABARAPL1 and GABARAPL2, the ATG8 family is central component of autophagy in most organisms, because of its role in autophagosome formation and maturation. During the autophagy process, LC3/GABARAP proteins are conjugated to phosphatidylethanolamine (PE) and lipidated forms are integrated into phagophores that will form mature autophagosomes after capture of cytoplasmic material and membrane fusion. LC3/GABARAP conjugation is a multi-step enzymatic reaction that is biochemically distinct from other UbLs involving specific enzymes such as the E3 complex ATG5/12/16 that lipidates the distinct members of this family^7^. Internal and external membranes of the autophagosome structure integrate LC3/GABARAP proteins that contribute to organize cargo recruitment. Decaping of LC3/GABARAP from the external membrane occurs before fusion with the lysosome^8^. In ubiquitin-dependent autophagy, substrates are first ubiquitylated, thereby building complex signals composed by various types of ubiquitin chains that label structures to be degraded^9^. Cytosolic autophagy receptors contribute to cargo recruitment to the autophagosomes by linking ubiquitylated cargoes and LC3/GABARAP that have been integrated to the autophagosome. Several autophagy receptors such as p62 (also known as sequestosome-1/SQSTM1), NBR1, Tax1BP1 (also called TRAF6-binding protein, T6BP), Optineurin (Optn) or NDP52, integrate the so-called Sequestrome-1-like receptors (SLRs) family^10^. SLRs carry both an ubiquitin binding domain (UBD) and a LC3 Interacting Region (LIR) that condition their interaction with ubiquitin and LC3/GABARAP proteins^11^. LIR regions display a core consensus sequences [W/F/Y]xx[L/I/V] with variable binding affinities to the hydrophobic pockets of the different LC3/GABARAP proteins^12^.

In mammalians, the cargo recruitment mechanism requires multiple complex steps including conjugation/deconjugation of ubiquitin chains and participation of 6 LC3/GABARAP family members, tens autophagy receptors, and almost hundred LIR-containing factors that play distinct, but not yet fully characterized, roles during selective autophagy events^11^. For many of the pathologies where autophagy appears to play an important role, it is still not clear how these molecular processes are de-regulated. ATG8/LC3 proteins have been extensively used as biomarkers to monitor autophagosome formation, localization and dynamics in physiology and pathology. ATG8/LC3 antibodies are widely used to analyze autophagy by immunofluorescence or by Western blot to assess protein levels and detect lipidated forms^13^. Fluorescently-labelled ATG8/LC3 are also widely used for the same applications and to monitor dynamics of autophagy^13^. Tandem probes such as RFP-GFP-ATG8 or GFP-ATG8-RFP-ATG8DG have been developed to follow autophagy regulated processes^14,15^. However, the overexpression of complex structures could generate artifacts if these interfere with specificity of cargo recognition/recruitment, making data difficult to interprete. In particular, it is well known that endogenous expression of distinct AGT8/LC3 molecules is different relative to cell types or stress conditions, highlighting the importance of promoters used to overexpress these probes. LC3 sensors have been recently proposed to overcome the limits of the actual tools. Using the affinities of LIRs toward the different ATG8s proteins, two groups developed fluorescent engineer peptides that can be used to monitor autophagy in an endogenous manner^16,17^. Peptides interacting with ATG8 molecules were isolated by phage display or based on already known LIRs. Both tools are relatively specific and can be used as sensitive fluorescent probes to monitor ATG8 proteins by immunofluorescence in mammalian cells.

For more than a decade, engineered chimeric peptides have been developed to investigate ubiquitin and ubiquitin-like (UbLs) proteins in an endogenous way^18–20^. These peptides are designed to combine tags for easy purification and Ubiquitin or UbLs binding domains displayed in tandem. With a strong affinity and specificity for specific UbL proteins, these capturing systems allow the identification of endogenous proteins even if expressed at low levels within the cell. Here, we developed new molecular traps to isolate, identify and characterize processes regulated by ATG8/LC3 proteins. Based on LIR consensus sequences artificially disposed in tandem and fused to GST, these tools capture recombinant ATG8/LC3 proteins in vitro and show strong affinity and specificity towards GABARAPs or LC3s proteins extracted from distinct cell lines. Using these tools, we managed to differentiate LC3s from GABARAPs proteins in mammalian cells, and LGG1 from LGG2 in *C. elegans*. We were also able to isolate Atg8 from *S. cerevisiae* in distinct autophagy activating conditions. More importantly, these new molecular traps can be used to pulldown LC3/ATG8 interacting proteins allowing unprecedented advances in the opportunity to characterize macromolecular molecular complexes involved in autophagy. Thus, our new ATG8/LC3 traps provide an excellent way to study autophagy through the capture of endogenous or tagged ATG8/LC3 forms in some of the most commonly used biological models.

## Results

### Development of LC3-traps

**Figure 1:**
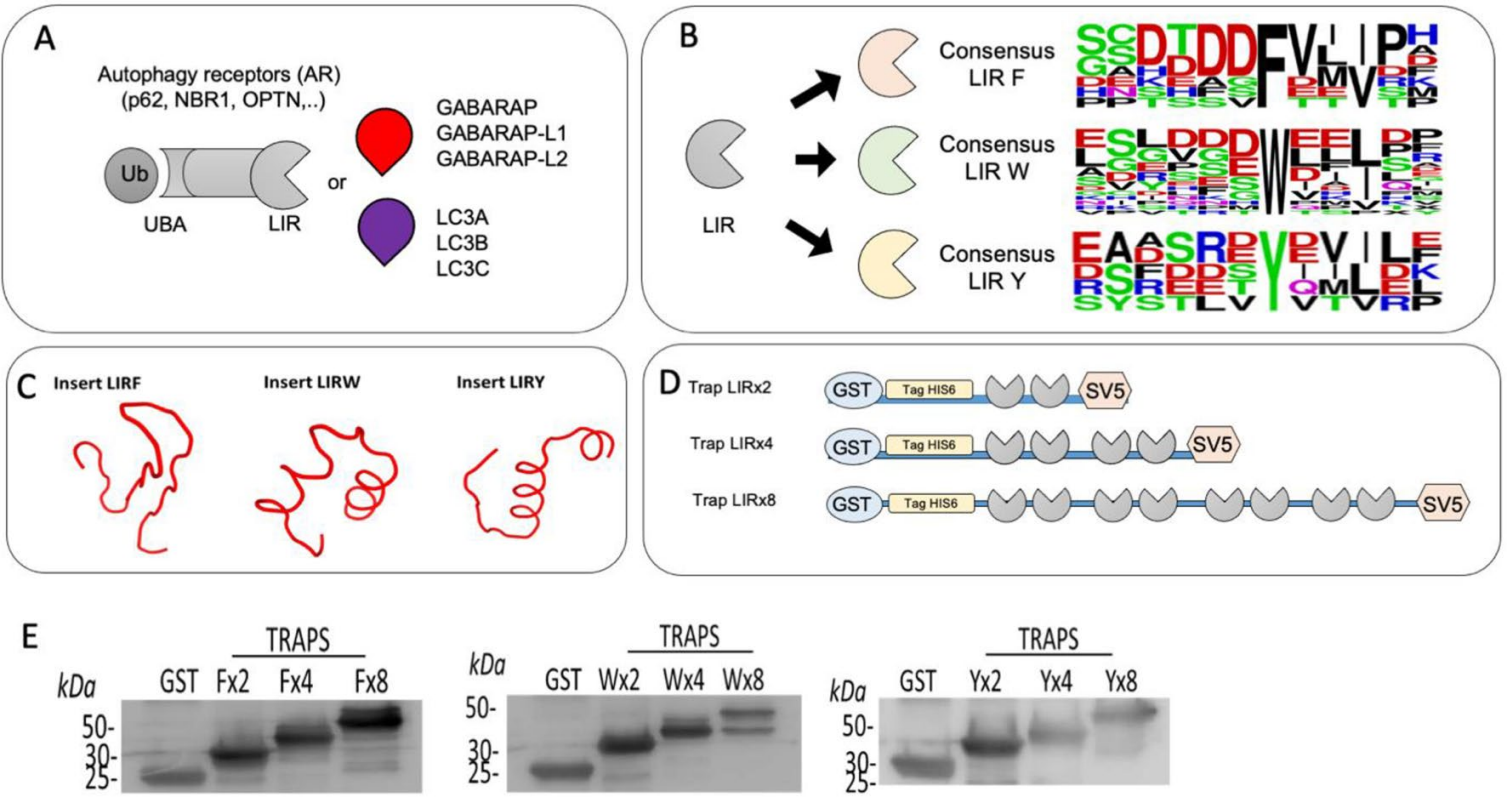
Strategy, design and production of LC3 traps. (**A**) Selective autophagy is driven by receptors that bind in one side ubiquitin chains through ubiquitin-binding domains (UBA) and in the other they interact with LC3/GABARAP proteins using interacting regions (LIR). (**B**) The three major consensus sequence were analysed using Weblogo software (https://weblogo.berkeley.edu) and named F, W or Y, according to the predominant hydrophobic amino-acid of these sequences. In the consensus, colours indicate chemical properties of amino acids: polar (G, S, T, Y, C, in green), neutral (Q, N, in purple), basic (K, R, H, in blue), acidic (D, E, in red), and hydrophobic (A, V, L, I, P, W, F, M, in black). (**C**) 3D structures of the peptides carrying two identical LIR consensus F, W and Y according to PEPFOLD software. Both LIR motifs face to each other in those 3D structures. (**D**) Representation of the molecular traps disposing 2, 4, and 8 LIR sequences in tandem. GST, His6 and SV5 tags are located at the N-terminus and C terminus of each construct. (**E**) Recombinant proteins produced in *E.coli*. Western blot analysis using anti-GST antibody of the distinct LC3-traps and GST control. Cleaved products of LC3-traps generated during the induction and purification procedure can be observed, in particular for traps containing 8 LIR repeats.

In order to isolate LC3/GABARAP containing autophagosomes, we consider the architecture of autophagy receptors that contain LC3-Interacting-Regions (LIR) (Fig. 1A). To increase the possibility to isolate distinct autophagosomes, our LC3-traps were designed in silico using weblogo to analyse known and putative LC3-Interacting-Regions (LIR) present in various proteins interacting with LC3/GABARAP^12^ (see “Methods”). LIRs are characterized by the presence of acidic amino acids surrounding hydrophobic patches of different extensions that help for the recognition of hydrophobic pocket of LC3/GABARAP proteins^12^. Three distinct LIR consensus motifs aggregate most of the sequences with the potential to interact with LC3/GABARAP proteins: LIR with a consensus F “FVII” like the one carried out by Optn, LIR with a consensus W “WEEL” like the one of SQSTM/p62 protein and LIR consensus Y “YDVI” present in NBR1 (Fig. 1B)^12^. The three LIR consensus sequences are 12 amino acid long and named according to the consensus hydrophobic amino-acids F, W and Y^12^. The cloned DNA inserts encoded 2 identical LIR sequences with a six nucleotides extension to generate a cleavage site compatible with BamH1 present in the pGEX6AP1-SV5 plasmid (see material and methods). Before generating these recombinant LIR proteins, their folding was predicted using PEPFOLD software^21^ (Fig. 1C). The folding of these peptides displayed the most probable 3D structures of each two LIR sequences. LIR dimers adopt an anti-parallel folding and is placed in front of the other in these unstructured peptides. In order to increase the affinity of these constructs for distinct LC3/GABARAP proteins, LIR dimers were cloned in tandem. For each of the three LIR motifs F, W and Y, 1, 2 and 4 inserts were cloned to generate traps with 2, 4 or 8 LIRs (Fig. 1D). Clones with correct DNA sequence were selected for protein production in *E. Coli*. Expression of these traps referred as Fx2, Fx4 and Fx8 for the LIR F-traps, Wx2, Wx4 and Wx8 for the LIR W-traps or Yx2, Yx4 and Yx8, for the LIR Y-traps were confirmed by WB with anti GST antibody (Fig. 1E).

### Validation and characterization of LC3-traps

**Figure 2:**
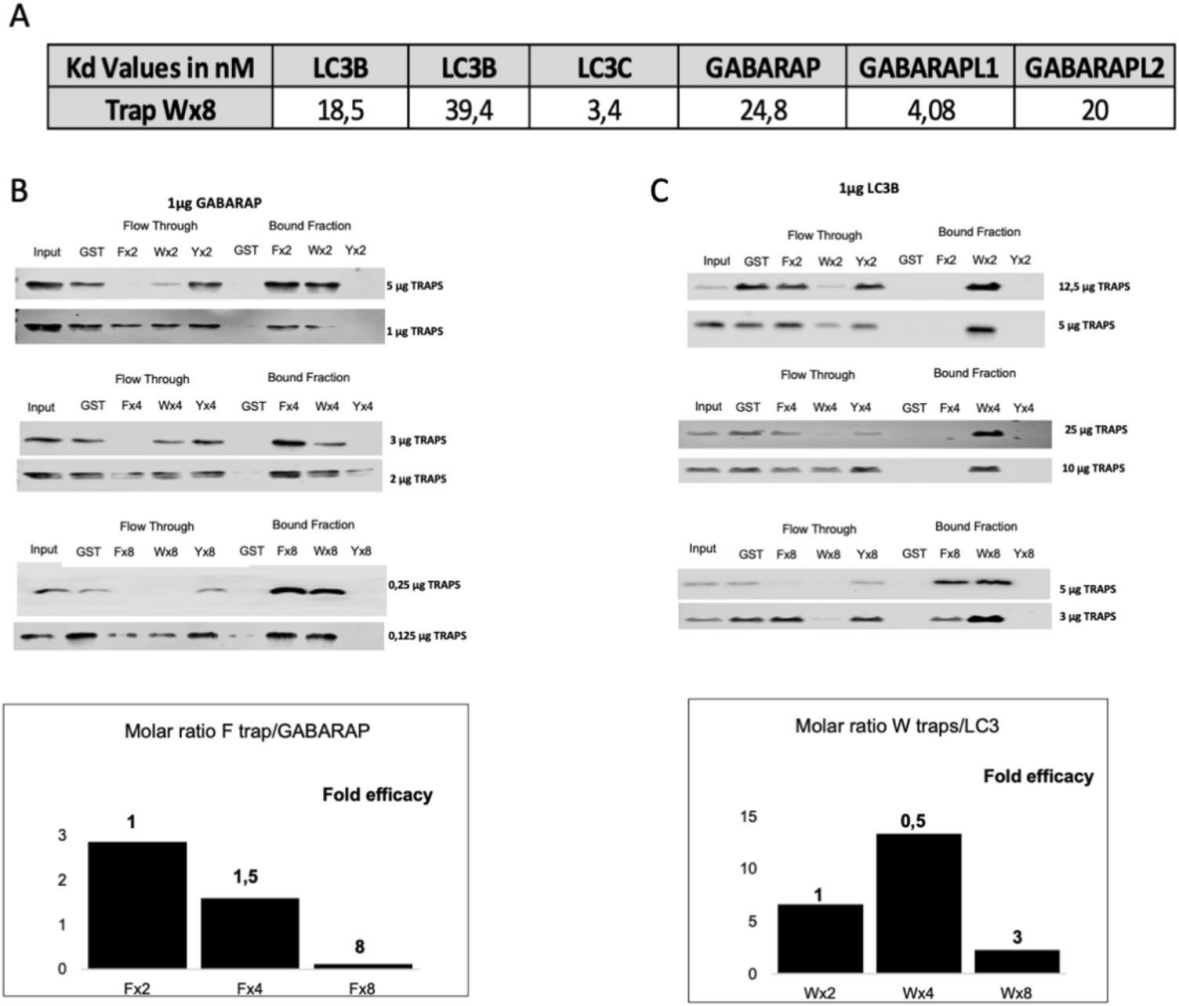
In vitro validation and characterization of LC3 traps. (**A**) Affinities between Wx8 traps with GABARAP or LC3 proteins were measured using microscale thermophoresis (MST). Kd values included in the table. Experiments were performed in duplicate. In vitro binding of GABARAP (**B**) or LC3B (**C**) with LC3-traps with 2, 4 or 8 LIRs using recombinant purified proteins. GST pulldown assays were used to analyze the capacity of distinct LC3-traps to capture recombinant LC3/GABARAP proteins. Various concentrations of LC3-traps were used to capture 1 mg of GABARAP or LC3B proteins. The graphs show the molar ratio used between trap and target as well as the fold efficacy between observed for traps with 2, 4 and 8 LIRs. Experiments performed twice. Input, Flow Through and Bound Fractions were analyzed by Western blot with specific antibodies against GABARAP or LC3B. Images of PVDF membranes were cut after antibody hybridization to optimize spaces.

Once the LC3-traps were produced in bacteria and purified, micro scale thermophoresis (MST) was used to evaluate their affinity towards LC3/GABARAP proteins. All traps were covalently labelled with an N-hydroxysuccinimide (NHS) dye and mixed with different concentrations of free LC3/GABARAP recombinant proteins. The time of binding response of the traps/LC3/GABARAP complexes in a temperature gradient (MST traces) was measured by fluorescence (Fig. 2A and Fig. S1). The fluorescent ligand-dose response plotted was a sigmoid curve where Kd values were fitted using the MO Affinity Analysis software (NanoTemper Technologies, Fig. S1). The affinities between LC3-traps Wx8 and all recombinant LC3/GABARAP proteins were measured and included in the table (Fig. 2A). The low/mid nanomolar range of the measured Kd reflects a strong affinity of the LC3-traps Wx8 in vitro towards all targets tested. To investigate if a cooperative binding effect, proportionally to the number of LIR domains included in each trap, could be observed in vitro GST-pull down experiments were performed with purified proteins. In these experiments, GABARAP or LC3B ligands were set to 1 μg and we used various concentrations of LC3-traps F, W or Y containing X2, X4 or X8 LIRs (Fig. 2B,C). Two criteria were considered to evaluate the efficacy of these traps: the amount of LC3/GABARAP proteins bound to the affinity columns, and the unbound material (Flow Through). With these data, we calculated the fold efficiency of these traps relative to the molar ratio used in each condition. Our results show that LC3B is captured with higher efficiency by W LC3-traps, while F LC3-traps show a small preference for GABARAP and confirm the cooperative binding effect associated to the number of LIRs present in each trap. Interestingly, LC3-traps Y only capture LC3/GABARAP proteins with a low efficiency in vitro, i.e. when LC3-trap Yx8 was used (Fig. 2B,C and Fig. S2). This could be interpreted as an enhanced capture efficiency associated to the multiple LIRs in a system where an excess of material is used.

**Figure 3:**
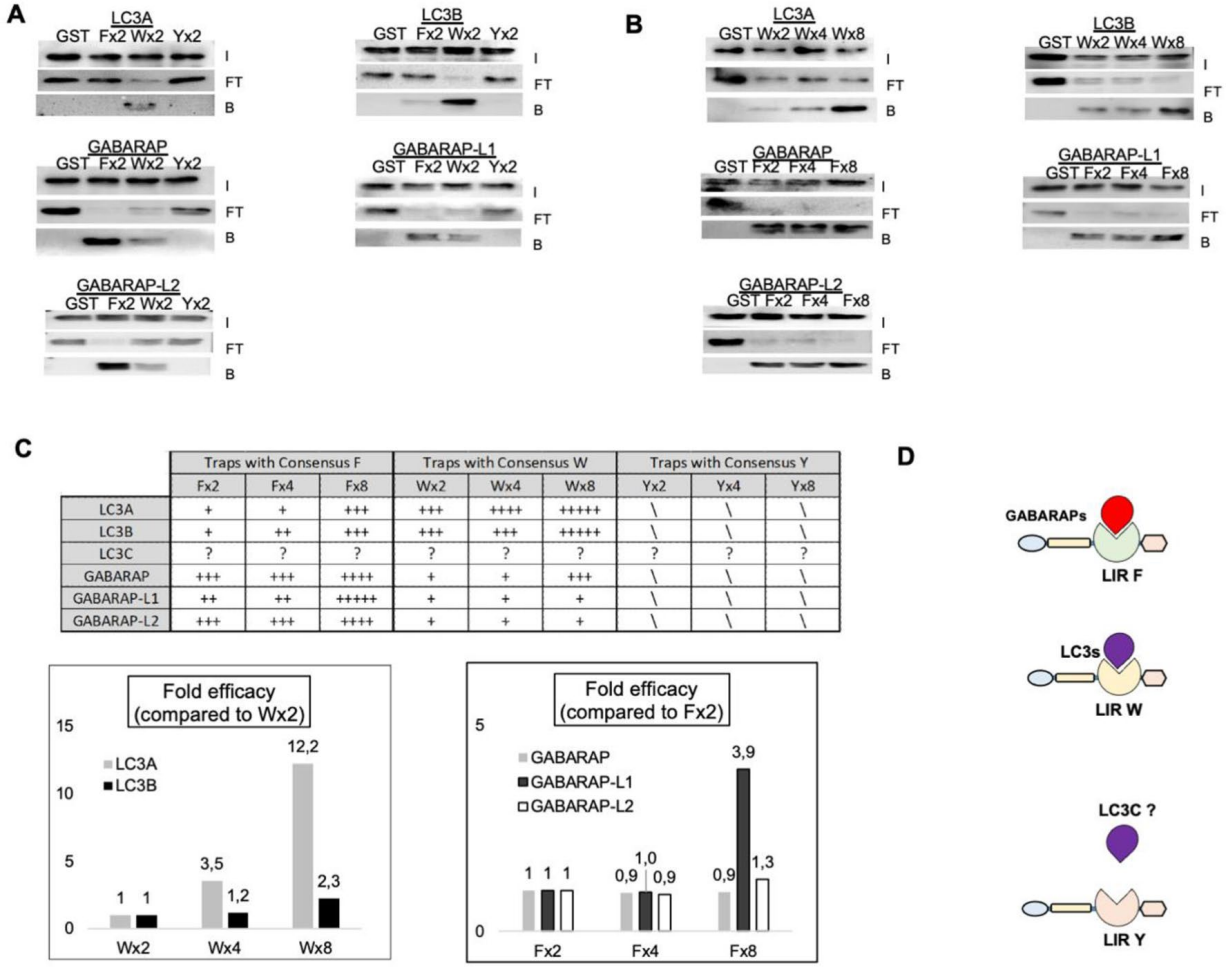
Cooperative effects and binding preferences of LC3 traps towards LC3/GABARAP proteins captured from cell extracts. **(A**) Cell extracts from twenty millions of ZBR cells treated 8 h with BafA were incubated 4 h with 100 µg of purified Fx2, Wx2, Yx2 LC3 traps. Captured material was analyzed by Western blot using specific antibodies against distinct LC3/GABARAP proteins as indicated. Input (I), flow through (FT) and bound (B) fractions are shown. (**B**) Using similar settings than in (**A**), 100 µg of purified LC3 traps carrying 2, 4 and 8 LIR motifs were incubated with ZBR cell extracts. Western blot analysis of the input (I), flow through (FT) and bound (B) fractions using specific antibodies for the indicated LC3/GABARAP proteins as indicated. These experiments were performed twice. Images of PVDF membranes were cut after antibody hybridization to optimize spaces. (**C**) Analysis of the results obtained from the capture obtained in A and B. Interactions are qualitatively annotated in the table as (+) low to (+++++) high interactions. No interaction is indicated as (\). Low levels of LC3C in these cells did not allow the detection of this protein in total cell extracts (Fig. S4). The lower panel shows the fold efficacy of distinct W and F Traps towards distinct LC3/GABARAP proteins, compared to traps contining 2 LIR domains. (**D**) Summary of the LC3/GABARAP proteins captured with distinct LC3-traps. Endogenous GABARAP proteins were preferentially captured by LIR-F traps, while LC3 molecules were best captured by LIR-W traps, except for LC3C that we could not evaluate. LIR-Y trap did not capture any of these proteins under the experimental conditions used.

To investigate the capacity of LC3-traps to capture endogenous LC3/GABARAP proteins, GST pull down experiments were performed using lysates obtained from ZBR mantle cell lymphoma cells that are resistant to the proteasome inhibitor. ZBR cells show a permanently activated autophagy, and in particular, a continuous degradation of proteasome subunits (proteaphagy)^22^ (Fig. 3). Results shown in Fig. 2 support the efficiency of the LC3-traps to capture recombinant LC3/GABARAP proteins. In vivo, these lipidated proteins are integrated into autophagosome membranes to endorse their specific roles during autophagy events. This lipidation state of the LC3/GABARAP proteins might change their availability and interaction with distinct partners, that could affect their recognition by the LC3 traps. To favor the accumulation of cellular factors targeted to degradation via autophagy, especially LC3/GABARAP proteins, ZBR cells were treated with Bafilomycin A (BafA)^22^. Under these conditions an active autophagy was observed and autophagy markers LC3B and p62 were accumulated (Fig. S3). To ensure the total capture of endogenous LC3/GABARAPs from 500 µg of total protein cell extracts, 100 µg of LC3-traps were used per pull down using similar conditions as previously reported^23^. After an overnight binding reaction, Input, flow-through and bound fractions were analyzed by Western blot using specific antibodies (Fig. 3A). Our results indicated that LC3-traps containing 2 LIRs, show different preferences for distinct LC3/GABARAP family members (Fig. 3A): while LC3-trap Wx2 preferentially interacts with LC3A and LC3B proteins, LC3-trap Fx2 more specifically binds GABARAP proteins, globally confirming what was observed when using purified recombinant proteins (see Fig. 2C). LC3-trap Yx2 did not pull down any of these proteins under the tested conditions. However, under the explored conditions expression levels of LC3C protein was not detectable in ZBR cells by Western blot in any fraction (Fig. S4). Importantly, ubiquitin was not found in the bound fraction, supporting the specificity of the LC3-traps towards LC3/GABARAPs proteins (Fig. S5). The multiplication of LIRs present in LC3-trap constructs up to 4- or eightfold repeated motifs showed cooperative binding effects of the capacity of LC3-trap W to capture LC3A and LC3B proteins and of LC3-traps F to capture GABARAP, GABARAPL1 and GABARAPL2 proteins (Fig. 3B). Table and schematic shown in Fig. 3C,D recapitulate the different capacities of the LC3-traps to bind LC3/GABARAP proteins purified from cell extracts.

### LC3-traps capture ATG8 orthologs in distinct biological models

**Figure 4:**
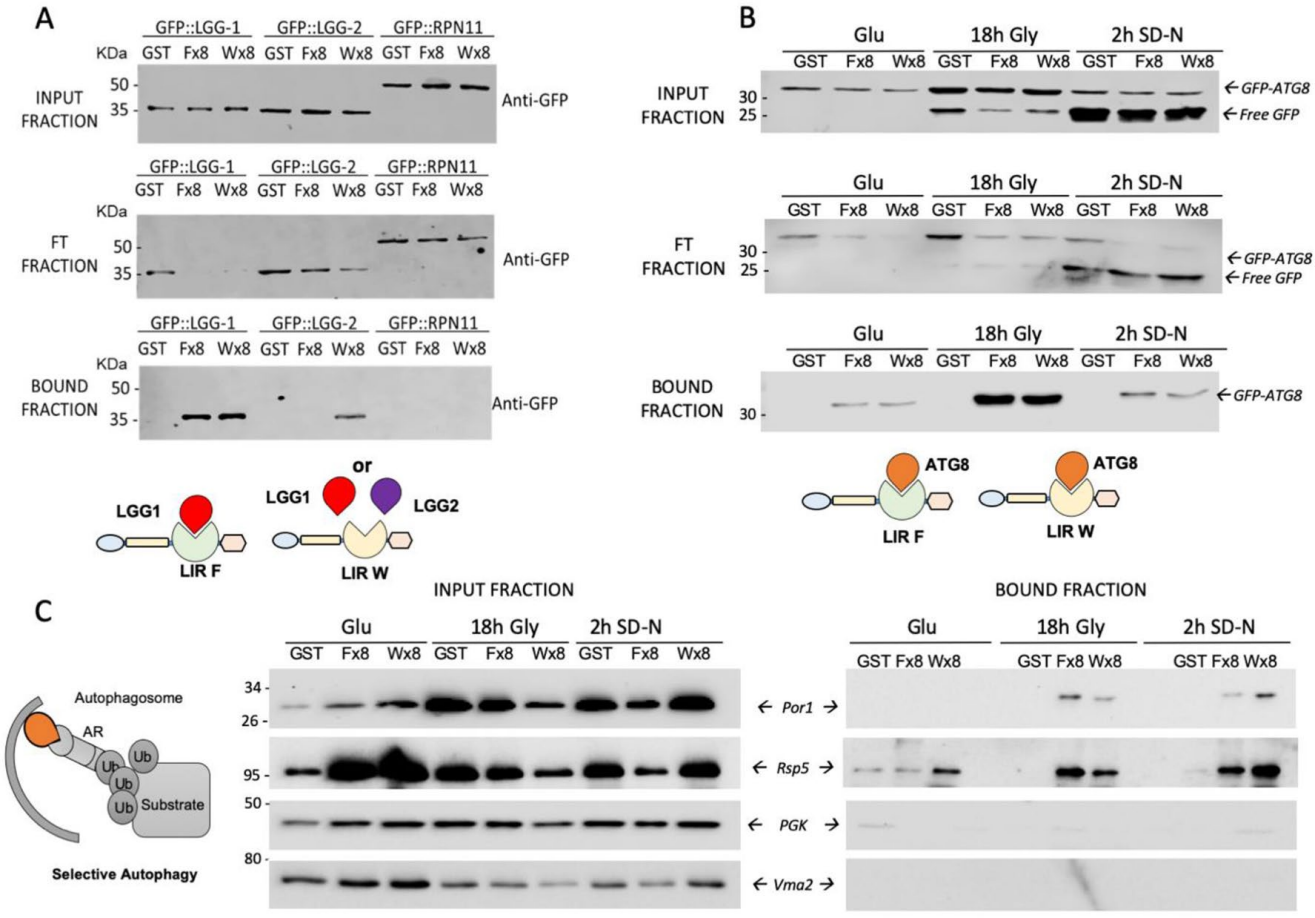
LC3 traps capture ATG8 orthologs in several biological models. (**A**) In vivo capture of LGG-1 and LGG-2 from *C. elegans* using LC3 traps. LGG-1 (GABARAP homolog) and LGG-2(LC3 homolog) were purified using tetrameric F and W LC3 traps from 3 different *C. elegans* strains as indicated (500 µg of total proteins from lysates were used per sample). GST-pulldown was performed as previously indicated and collected fractions were analyzed by Western blot using an anti-GFP. (**B**) Clones expressing GFP-Atg8 were cultured in glucose containing medium (glu), glycerol containing medium for 18 h (18 h Gly) or during 2 h in nitrogen depleted medium (2 h SD-N). One milligram/point of total protein yeast extracts from were incubated with Fx8, Wx8 LC3 traps or GST control. GST pulldown was performed as previously indicated and collected fractions were analysed by Western blot using an anti-GFP. (**C**) LC3-trapped fractions were analysed for the presence of the mitochondrial membrane protein Por1, suggestive of mitochondrial sequestering in autophagic compartments. The presence of other proteins (Rsp5, PGK, Vma2) in the different fractions was tested by Western blot analysis. PVDF membranes were cut before antibody incubation to be able to detect several proteins from the same membraine.

To investigate whether these tools can be used to explore ATG8-regulated events in other organisms, we tested our LC3-traps in *C. elegans and S. cerevisiae*, two model organisms widely used to study autophagy. In contrast to mammalian cells, *C. elegans* cells express only two members of the ATG8 family, LGG-1 (GABARAP homolog) and LGG-2 (LC3 homolog)^24^. To study basal levels of autophagy, pull-down assays were performed with LC3-traps Fx8 and Wx8 in cell lysates from three different *C. elegans* strains, one stably expressing GFP-LGG-1, a second expressing GFP-LGG-2 and a third one expressing GFP-RPN11 that was used to control non-specific binding of these LC3-traps (Fig. 4A). Input, flow through and bound fractions were analyzed by Western blot using anti-GFP antibodies. Our results indicated that both LC3-traps Fx8 and Wx8 interacted with LGG-1, while only LC3-trap Wx8 interacted with LGG-2 (Fig. 4A) recapitulating the differential characteristics of binding observed using purified mammalian LC3/GABARAP proteins (Fig. 3). Interestingly, LC3-traps F and W capture the entire pool of expressed GFP-LGG-1, since this protein was undetectable in the FT fraction (Fig. 4A). In contrast, LGG-2 was only partially absent from the FT fraction of the LC2-trap W, suggesting binding preferences for this trap. GFP-RPN11 proteins were not found in the bound fraction, supporting the specificity of the traps towards their LGG-1 and LGG-2 targets. We have performed similar experiments in *S. cerevisiae* that encodes a single ATG8 molecule. For yeast experiments, we chose to express a GFP-Atg8 fusion protein since its cleavage can be used to monitor autophagy mediated degradation when using growth conditions that are known to activate autophagy^25^. Indeed, when autophagy is induced, GFP-Atg8 is cleaved in the lysosome/vacuole and since GFP is transiently resistant to proteolysis, the accumulation of free GFP is a good readout of autophagy completion. Three conditions of yeast culture were used. Yeast cells were first cultured to the exponential phase in a fermentative medium (Glu: Glucose), and shifted for 18 h in a respiratory medium containing glycerol (18 h gly) to increase mitochondrial mass and induce stationary phase mitophagy. Cells were then transferred for 2 h in a nitrogen depleted medium supplemented with glucose (SD-N), a condition known to induce mitophagy^26^. Input, flow through and bound fractions were analyzed by Western blot using an anti-GFP antibody (Fig. 4B). Yeast cells grown to the exponential phase in fermentative conditions show low levels of GFP-Atg8 and undetectable level of autophagy (no free GFP in the Glu input fraction). GFP-Atg8 expression and autophagy were induced in respiring cells grown to the stationary phase (Input fraction, 18 h gly). Massive autophagy (mitophagy) occurred in nitrogen depleted conditions (Input fraction, 2 h SD-N). Our results indicated that both LC3-traps capture GFP-Atg8, in all tested conditions (Fig. 4B). Since we used conditions that induced mitophagy, we investigated if mitochondrial proteins could be detected under these conditions. The Western blot analysis of distinct fractions from the same experiment showed that the outer mitochondrial membrane protein Por1/VDAC can be captured showing that these conditions allow the capture of autophagosomes containing mitochondrial membranes (Fig. 4C). Interestingly, the HECT ubiquitin ligase RSP5 was captured in our conditions (Fig. 4C). A role for Rsp5 in yeast mitophagy has been previously proposed^27^. Our results show that Rsp5 is closely interacting with mitochondrial proteins and/or Atg8, and that this interaction occurs mostly after mitophagy induction (Fig. 4C). As negative controls, we tested the cytoplasmic soluble PGK (phosphoglycerate kinase) and the vacuolar protein Vma2 that show absence or non-specific capture.

### LC3-traps capture selective autophagy cargoes

**Figure 5:**
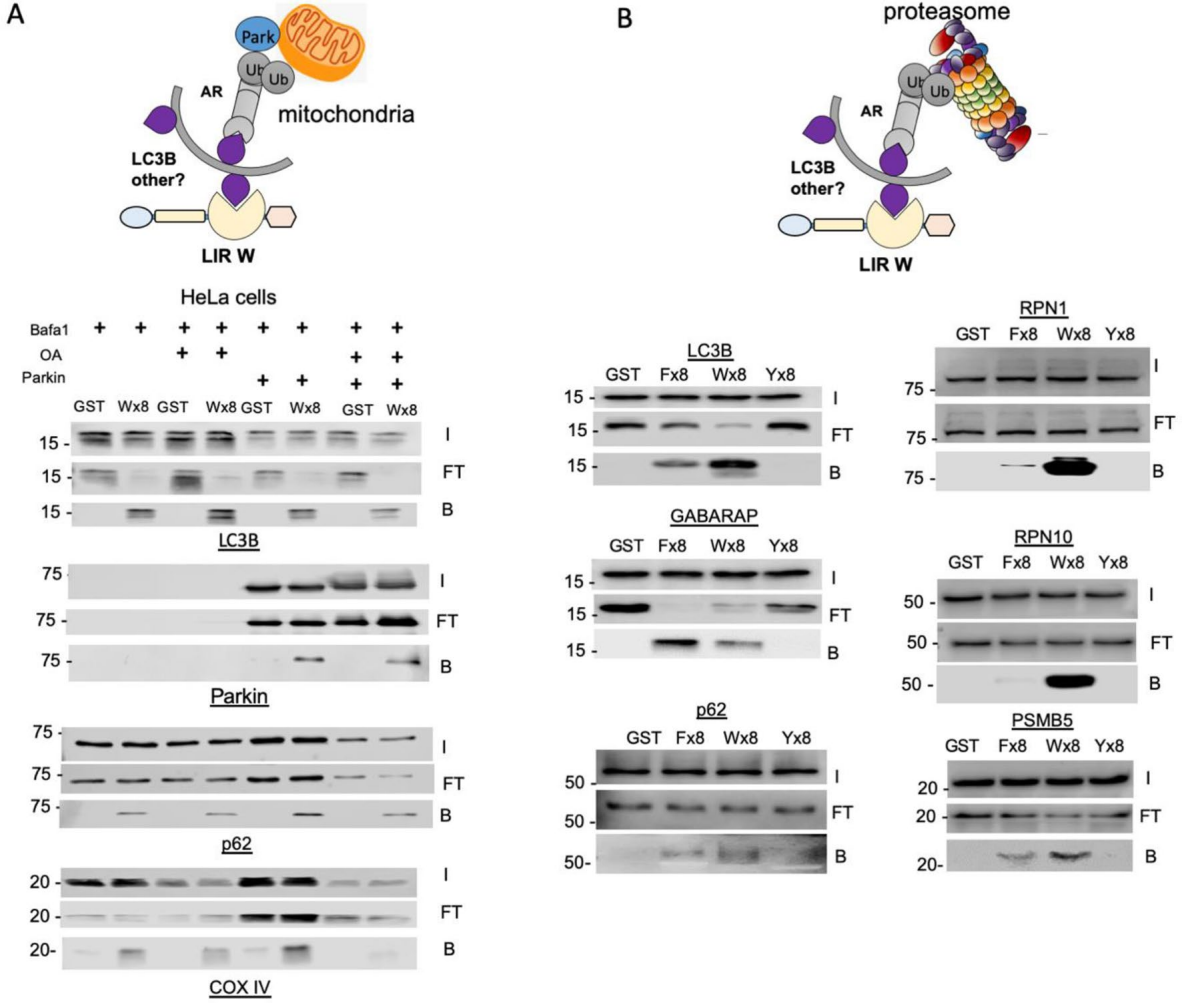
LC3 traps isolate substrates degraded by distinct selective autophagy pathways. (**A**) Analysis of parking induced mitophagy in HeLa cells transfect or not with parking and treated with Bafilomycin A (Bafa 200 nM) or Bafa + Oligomycin/Antimycin (200 nM + 10 µM each). One milligram of total protein from these lysates were incubated 4 h with 100 µg of either GST control or Wx8 traps. Input (I), flow-through (FT) or bound (B) fractions were analysed with specific antibodies for the indicated proteins including mitochondria membranes protein COX IV and mitochondria associated protein Parkin. (**B**) Analysis of the permanently activated proteaphagy present in ZBR cells. Capture of proteasomes subunits was performed using Fx8, Wx8 or Yx8 LC3 traps. 500 µg of total protein lysates from ZBR cells treated with BafA were incubated 4 h with each LC3-trap. Input (I), flow through (FT) and bound (B) fraction were analysed by Western blot with the indicated specific antibodies. PVDF membranes were cut before antibody incubation to blot for several proteins.

Autophagy receptors selectively brings together cargoes into autophagosomes for lysosomal degradation. Cargoes such as mitochondria or proteasome interact with one or more autophagy receptors that recognize one or several LC3/GABARAP proteins. To investigate if our LC3-traps could be used to isolate specific cargoes driven into autophagosomes, we used different conditions to explore mitophagy and proteaphagy in mammalian cells. Parkin-mediated mitophagy was studied using a well-known HeLa model in which this ubiquitin E3 is expressed to drive autophagy-lysosomal dependent degradation. HeLa cells were also stimulated with OA (Oligomycin A/Antimycin A1) to induce mitophagy and co-treatment with Bafilomycin A (BafA) was used to block lysosomal degradation. In mammalian cells various autophagy receptors have been implicated in mitophagy including Optn, NDP52 and p62^10,28,29^. Deletion of a single receptor appears to be partially compensated by another, highlighting their functional redundancy^30^. Since p62 contains a W-LIR motif, we used the W LIR-trap^12^. Total cell extracts were incubated with purified LC3 traps with Wx8 LIR motifs. Input, Flow through and bound fraction were analyzed by Western blot (Fig. 5A). Our results indicate that LC3B is efficiently captured in all conditions tested. Both autophagy receptor p62 and the mitochondrial protein COX IV were bound to Wx8 LC3-trap (Fig. 5A). Following OA induction, degradation of both proteins by the autophagy process, observed in Input conditions, results in reduced binding by LC3-Traps. Interestingly, Parkin is also efficiently captured by Wx8 LC3-trap when expressed, indicating that during the ubiquitin-dependent mitophagy, not only LC3-binding but also cargoes-associated proteins could be captured by LC3-traps. Results obtained in yeast and HeLa cells therefore indicate that LC3-traps can be used to explore mitophagy in various biological models.

To investigate if proteaphagy could be studied using our LC3-traps, we used BTZ resistant MCL cells where permanently activated autophagy has been observed^22^. In this model, p62 appears to play an important role in the recruitment of proteasomes into autophagosomes^22^. The participation of p62 was confirmed since it was captured in different proportions by all LC3-traps. However, the LC3-trap WX8 is the one capturing this autophagy receptor with the highest efficiency (Fig. 5B). As previously observed, Fx8 traps preferentially bind to GABARAP proteins, while Wx8 traps show preference towards LC3B protein. Interestingly, a strong signal of RPN1, RPN10 and β5 (PSMB5) was observed in the bound fraction to Wx8 LC3-traps (Fig. 5B), indicating that the Wx8 traps was the best molecular trap to study proteaphagy. Fx8 LC3-trap captured with lower efficiency the proteasome subunits suggesting that if any other autophagy receptor with a F-LIR consensus could participate in proteaphagy, its role might not be preponderant. Although our results do not exclude the participation of other autophagy receptors with the same W-LIR motif than p62, our observations highlight a major role of p62 in proteaphagy.

## Discussion

Understanding the intricate mechanisms of autophagy regulation is critical to improve current clinical treatments for pathologies where this proteolytic pathway appears to play a crucial role. Autophagy selectivity during cargo recruitment relies on a complex combination of several post-translational modifications involving distinct ubiquitin family members, enzymatic activities, autophagy receptors and cofactors. In particular, the role of distinct LC3/GABARAP proteins that in yeast appears to be achieved by a single ATG8 molecule. Although the distinct role of LC3/GABARAP proteins in the autophagy recruitment is under deep investigations, tools to isolate and purify these proteins remain limited. Furthermore, autophagy signaling is finely regulated and overexpression or incorporation of complex tags can generate artifacts when studying the role of distinct LC3/GABARAP proteins. This underlines the necessity to study these molecules at endogenous levels as the observations will be more accurate and provide more physiologically relevant information.

In this study, we developed new molecular traps to isolate LC3/GABARAP proteins. Our traps display multimeric LIRs and can be used to capture endogenous LC3/GABARAPs in various biological models. We used three different LIR consensus with predominant hydrophobic amino-acids F, W and Y found in the autophagy receptors Optn, p62 and NBR1 respectively. These LIRs are known to display affinity and specificity towards one or several LC3/GABARAP family members. The GST tag of our LC3-traps was used to pull down LC3/GABARAP interacting proteins and other cellular factors directly or indirectly associated to these UbLs in mammalian cells or equivalent molecules in *C. elegans* or *S.cerevisiae * models.

Our traps showed binding preferences towards LC3/GABARAP proteins. For instance, the traps carrying the LIR F, mimicking that of Optn, revealed preferential affinity for GABARAP, GABARAP-L1 and GABARAP-L2 proteins in mammalian cells, and for LGG1 in *C. elegans* model. The traps carrying the consensus LIR W, present in p62, displayed higher affinity LC3A and LC3B in mammalian cells and LGG2 in *C. elegans*. LC3C was not expressed in the cellular models used and could not therefore be evaluated for its capacity to be captured by traps. The traps carrying the consensus LIR-Y showed low in vitro interactions with recombinant LC3 or GABARAP.

Our LC3 traps show strong affinities in vitro (low and mid nanomolar range) and specifically capture endogenous levels of LC3/GABARAP proteins. We also demonstrated that these molecular traps can be used to explore two distinct selective autophagy events, mitophagy and proteaphagy. The use of LC3-Traps with mitophagy cell models similar to the one used here could be undoubtedly usefull to explore the roles of distinct autophagy receptors in this complex proteolytic event. Our LC3-traps capture proteasome subunits in conditions where autophagy is permanently activated. In this work, we could show for the first time,  involvement of LC3 proteins rather than GABARAP proteins in the recognition of proteasome in proteaphagy. Using these molecular traps, it could be interesting to explore if other selective autophagy events are simultaneously occurring under the explored conditions and what is the interconnection between them. Altogether our data indicate that these new molecular traps are interesting tools to study the regulation of selective autophagy events.

Considering the properties of the presented LC3-traps, these tools can most likely be used as sensors to follow autophagy flux^16,17^. Since the mechanism of action of our LC3-traps is based in evolutionary preserved functional interactions, these work in all tested species. This represents a major advantage of our LC3-traps with respect to the available antibodies since antibodies don’t work with all species due to the low immune-reactivity of the well-preserved epitopes. For instance, in the absence of commercially available antibodies for LGG-1 and LGG-2 we have used tagged versions for both molecules.  Endogenous forms of LGG-1 were also captured with our LC3-traps and confirmed by mass spectrometry (Legouis et al., personal communication). Another particularity and advantage of our traps is that they can be used to characterize in vitro molecular interactions or quantitative or HST methods to screen inhibitory molecules as it was the case for other molecular traps that capture ubiquitin (TUBEs) or SUMO (SUBEs)^19^. Since the capture efficiency of LC3/GABARAP proteins is high and interacting proteins are easily detected, these tools have the potential to be used in mass spectrometry studies to better understand selective autophagy events.

## Methods

### Bioinformatic analysis of LIRs

The three LIR consensus sequences used to design 3 different types of LC3-traps are 12 amino acid long and named according to their consensus F, W and Y^12^. Seventeen W-LIR, 7 F-LIR or 5 Y-LIR sequences were analyzed using Weblogo software (https://weblogo.berkeley.edu) that computes sequence logo after multiple sequence alignments. In this graphical representation, the overall height of the stack traduces the sequence conservation at that position, while the height of symbols within the stack indicates the relative frequency of each amino or nucleic acid at that position. In all sequence logo created from sequences of the same consensus, the amino acid phenylalanine (F), tryptophan (W) and tyrosine (Y) of the three LIR core motifs were represented with 100% relative frequency, as expected. The most represented amino acids in the alignment performed with Weblogo were selected for the LIR sequences that were used to design the LC3-traps.

The cloned DNA inserts used for building the LC3-traps encoded 2 identical LIR sequences according to the Weblogo alignment. Between two inserts a small linker of two additional amino acid GS were located at the C terminal. These amino acids were a requisite to create cloning sites to be inserted into pGEX6AP1 cloning plasmid (see below). The folding of these peptides was predicted using PEPFOLD software^21^ that displayed the most probable 3D structures of the peptide encoded by the designed sequence. Each LIR adopts an anti-parallel folding and is placed in front of the other in these unstructured peptides.

### Western blotting

Mammalian cell extracts were lysed in 1.5X Boiling Buffer (4% SDS, 20% glycerol, 120 mM Tris–HCl pH 6.8). Cell lysate were loaded onto 8%–15% acrylamide gels. Acrylamide concentrations were chosen depending on the size of the analyzed proteins. Proteins were electrophoretically separated at 100 V during 5 min in Laemmli Buffer for SDS PAGE (25 mM Tris, 192 mM Glycine, 0.1% SDS, pH 8.6), then at 140 V until the migration front reached the bottom of the gel. Protein wet-transfer were performed onto PVDF membrane (0.45 µm pore size, Immobilon-P, Merck) in buffer containing 20% ethanol and 80% 1 × transfer buffer (transfer buffer 10x: 240 mM TRIS, 2 M Glycine), either for 2 h (240 mA per gel at 4 °C) or overnight (32 mA per gel, 4 °C). Membranes were blocked for 1 h with either 5% milk or 5% Bovine Serum Albumine (BSA) (Sigma) diluted in 1 × Tris-buffered saline (TBS). Membranes were incubated with appropriate primary antibodies overnight at 4 °C. Secondary antibodies were added for 1 h at room temperature (RT) in blocking solution or in TBS only. Membranes were washed 3 times 10 min with TBS after incubation with primary and secondary antibodies. Pictures were acquired using West Femto ECL (34096 Thermofischer), with PXI4 (Syngene), GeneTools (Syngene) or Odyssey Fc (LICOR). Quantifications were performed using ImageJ.

For fluorescent imaging of Western blot membranes, 0.45 µm pore size Immobilon-FL were used (Merck). Secondary fluorescent antibodies were diluted in blocking solution supplemented with 0.2% Tween (Sigma) and 0.01% SDS. Antibodies washes were performed with TBS-Tween 0.1%. Before imaging, membranes were dried in the dark for at least 1 h.

### Cloning

Single DNA brands were generated by Eurogentec. Oligos were designed to expose in 5’ BglII and BamHI in 3’ for a direct cloning into the BamHI site of the pGEX6AP1 vector (see annex 1). These LIR coding sequences were ligated with themselves to generate multiple insert sizes. The previously digested and purified vector was directly added to the ligation mix containing the pool of LIR sequences. These two ligation steps were performed separately for each of the three LIR sequences. After DNA precipitation/concentration, the three ligation reactions were transformed in electrocompetent DH5α cells. Fourty positives clones for each of the three LIR-traps were selected for a total of 120 screened clones. As the plasmid contained the antibiotic (Ampicillin) resistance, colonies were selected and maintained in LB media with this antibiotic. The PCR screening gave information on the number of LIR inserts cloned into the pGEX plasmid, according to the size of the amplified fragment. PCR positive clones containing one, two or 4 inserts were transformed in chemocompetent BL-21 cells, for protein expression by induction with IPTG. Cell lysate from those cultures were analyzed by Western blotting with anti-SV5. The positive clones expressing the SV5 tag were sent for DNA sequencing. For each of the three LIR motifs, three traps with 1, 2 and 4 LIR cloned inserts were selected for a larger protein production (see below).

### Protein expression and purification

The LC3 traps were expressed in *E. coli* BL-21 strain and purified by affinity chromatography using a glutathione agarose matrix as previously reported^20,31^. All proteins were expressed in Escherichia coli BL-21 withinwith IPTG induction (1 mM) for 16 h at 20 °C. Cells were lysed by sonication in Lysis buffer (PBS, 0.5 M NaCL, 2 mM benzamidine, 1 mM PMSF) on ice (6 times 30 s pulses) and supplemented with 1% Triton. After 2 h centrifugation at 20,000 r.p.m., supernatant was subjected to standard glutathione agarose affinity purification (Sigma-Aldrich, St Louis, MO, USA) according to the manufacturer’s instructions. Samples were dialyzed using 12 kDa tubing (Sigma-Aldrich, St Louis, MO, USA) and concentrated at 2 μg/μL using Amicon Ultra with 3 KDa MWCO (Millipore). Purified LC3 traps were quantified by Nanodrop (280 nm absorbance) and supplemented with 10% glycerol before storing at − 80 °C.

### Cell culture

Mantle Cell Lymphoma Z-138 was obtained from the ATCC collection. ZBR was derived from Z-138^22^. Both cells were cultured in RPMI 1640 with 2 mM l-glutamine, 100 Units/mL penicillin, 100 µg/mL streptomycin and 10% fetal bovine serum (FCS). Cells were incubated at 37 °C, 5% CO_2_. Cell lines authentication was based on short tandem repeat (STR) profiling by DSMZ services (Braunschweig, Germany).

#### HeLa cells and mitophagy induction

HeLa cells were purchased from the ATCC collection (CCL-2™). HeLa stably expressing Parkin, were generated by stably transfecting EBFP2-Parkin expressing plasmid (kind gift from Prof Wei Yuan Yang, Institute of Biochemical Sciences, Taiwan). Cells were grown in DMEM supplemented with 100 Units/mL penicillin, 100 µg/mL streptomycin and 10% fetal bovine serum (FCS). HeLa clones stably expressing BFP-tagged Parkin were selected with 200 μg/mL of Geneticin (G418) and positive clones sorted by FACS. For mitophagy induction, cells were preincubated for 1 h with 200 nM bafilomycin A1 followed by treatment with cells with 10 μM of Oligomycin A and Antimycin A (OA) for 3 h in the continuous presence of Bafilomycin A1. Cells were washed twice in PBS 1X and harvested before lyse as previously described.

#### *C. elegans* culture and strains

Nematode strains were grown on nematode growth media (NGM) plates (autoclaved 1.5 g NaCl (Sigma-Aldrich, 60142), 1.5 g bactopeptone (Becton–Dickinson, 211677), 0.5 mL cholesterol (Sigma-Aldrich, C8667) 5 mg/mL, 1L H2O, 10 g bacto agar (Becton–Dickinson, 214010) supplemented with 500 µL CaCl_2_ (Sigma-Aldrich, C3306) 1 M, 500 µL MgSO_4_ (Sigma-Aldrich, M5921) 1 M, 10 mL KH_2_PO_4_ (Sigma-Aldrich, P5655) 1 M, 1650 µL K_2_HPO_4_ (Sigma-Aldrich, 60356) 1 M and fed with *Escherichia coli* strain OP50 (Caenorhabditis Genetic Center, E. coli HT115[DE3; https://cgc.umn.edu/). Strains used were the wild-type N2, DA2123 (*gfp::lgg-1*), VIG-9 (*gfp::lgg-2*) and *RPN-11::gfp*. Total protein extracts were prepared from a population of 4th larvae and adults. The worms were collected by centrifugation, and mixed with the TUBE lysis buffer containing glass beads (SIGMA G8772-100G). Two cycles of grinding (6000 rpm, 1 min) were done using Precellys 24 machine and extracts were frozen before capture experiments.

#### Yeast cultures

Wild type yeast cells (DF5, Mata, his3-∆200; leu 2–3, 2–112; lys2-801; trp1-1 (am); ura 3–52) transformed with the pRS416-GFP-ATG8 (CEN, URA3) plasmid (Kind gift from Fulvio Reggiori) were grown at 30 °C in YP (1% yeast extract, 2% peptone) or in minimal medium YNB (0.67% yeast nitrogen base without amino acids) (BD Difco) supplemented with a 0.1 g/L mixture of each amino acid and nucleic acid base component (Sigma-Aldrich), except URA for plasmid selection. The carbon source was 2% glucose or 2% glycerol. Before mitophagy induction, yeast cells were first grown in glucose containing medium and then shifted to glycerol containing medium for induction of respiration and increase of mitochondrial mass. Mitophagy was induced by further growth in glycerol containing medium to the stationary phase or by shifting the cells to nitrogen depleted medium (SD-N) with glucose as a carbon source. In this medium, cells undergo autophagy for nitrogen recovery and since mitochondria are not necessary in fermentative conditions (glucose containing medium), mitophagy is favoured over the other forms of autophagy.

### Pull down/capture

Pulldown experiments were performed essentially as previously described^23^ with minor modifications as indicated below.

For in vitro capture experiments, various amounts of purified trap were used to pull down 1 µg of recombinant LC3s proteins, in PBS star binding buffer [PBS 1X, 0.1% Igepal, 2 mM DTT]. Traps and free LC3s recombinant proteins were incubated 1 h rotating at 4 °C with Gluthatione-S transferase beads (Sigma).

For in vivo pull down, twenty millions ZBR cells treated 8 h with 20 nM BafilomycinA (or 500 µg of total proteins from *C. elegans* lysate) were incubated on ice 10 min in TUBE lysis buffer [50 mM sodium fluoride, 5 mM tetra-sodium pyrophosphate, 10 mM β-glyceropyrophosphate, 1% Igepal CA-630, 2 mM EDTA, 20 mM Na_2_HPO_4_, 20 mM NaH_2_PO_4_, and 1.2 mg/mL complete protease inhibitor cocktail (Roche, Basel, Switzerland)] supplemented with 100 µg of the different LC3 traps (containing one, two, or four LIR domains) or GST, used as a control. After cold centrifugation at 16,200*×*g for 30 min, supernatant was harvested. Lysates were added to 200 µL of prewashed/binding buffer equilibrated glutathione-agarose beads (Biontex, Martinsried, Germany), either for 6 h or overnight at 4 °C. Beads were centrifugated, 1000×*g* for 5 min (Beckman Coulter Microfuge 22R, Fullerton, CA, USA), and 1/10 of the unbound fraction or flow-through (FT) was saved for Western blot analysis. Three to four washes were carried out using 10 column volumes of wash buffer (PBS-tween 0.05%). Sample elution was done in 100 µL of Laemmli Buffer 3X.

### Micro scale thermophoresis (MST)

Thermophoresis was performed with a Monolith NT.115 machine (Nanotemper). LC3 traps were previously dyed covalently using the Monolith Protein Labeling RED-NHS 2nd Generation kit from Nanotemper. Affinities were measured between the NHS-dyed traps and each of the LC3s recombinant protein. To determine each Kd, LC3s proteins were diluted to prepare 16 samples at different concentrations, and placed with a constant concentration of fluorescent traps (20 nM). LC3A was used to determine MST within dilution from 6.5 µM to 0.2 µM, LC3B from 3 µM to 91 nM, LC3C from 1 µM to 30 nM, GABARAP from 60 µM to 1.8 µM, GABARAP-L1 from 12 µM to 0.36 µM, GABARAP-L2 from 20 µM to 0.6 µM. The interaction partners were mix and measured after equilibrium in binding buffer. Binding buffer used for traps-LC3A and traps-GABARAP L2 interactions: 25 mM Hepes pH8.0, 90 mM NaCl, 5% Glycerol, 0.5 mM DTT, 0.05% Tween, 1.85 mM KCl, 5 mM Na_2_HPO_4_, 0.9 mM KH_2_PO_4_. Binding buffer used for traps-LC3B and traps-GABARAP L1 interactions: 10 mM Tris HCl pH8.0, 115 mM NaCl, 5% Glycerol, 0.5 mM DTT, 0.05% Tween, 1.85 mM KCl, 5 mM Na_2_HPO_4_, 0.9 mM KH_2_PO_4_. Binding buffer used for traps-GABARAP interactions: 25 mM HEPES pH8.0, 90 mM NaCl, 2.5% Glycerol, 0.5 mM DTT, 0.05% Tween, 1.85 mM KCl, 5 mM Na_2_HPO_4_, 0.9 mM KH_2_PO_4_. Binding buffer used for traps-LC3C interactions: 10 mM Tris HCl pH8.0, 90 mM NaCl, 10% Glycerol, 0.05% Tween, 1.85 mM KCl, 5 mM Na_2_HPO_4_, 0.9 mM KH_2_PO_4_. Samples were loaded using Monolith NT.115 Premium Capillaries (Nanotemper), temperature was stabilized at 22 °C and excitation laser power set at 60%. Time response to temperature gradients were measured and Kd values were fitted to curve responses using the MO Affinity Analysis software (Nanotemper).

### Quantification and statistical analysis

All experiments were repeated for at least 3 times unless stated in the Figure legend. Two-tailed unpaired Student’s t tests were applied for comparisons between two groups. The data are presented as the means ± SD except stated otherwise. The *p < 0.05, **p < 0.01 or ***p < 0.001 values were considered statistically significant.

## Supplementary Information

Supplementary Information 1.

Supplementary Information 2.
