# Supplementary material for: Exploring selective autophagy events in multiple biologic models using LC3-interacting regions (LIR)-based molecular traps
Source: Sci Rep. 2022 May 10;12:7652. doi: 10.1038/s41598-022-11417-z (PMC9090809; doi:10.1038/s41598-022-11417-z)
Supplement: Supplementary file 1 — Supplementary Information 1. [file 41598_2022_11417_MOESM1_ESM.pdf]

Figure 1E

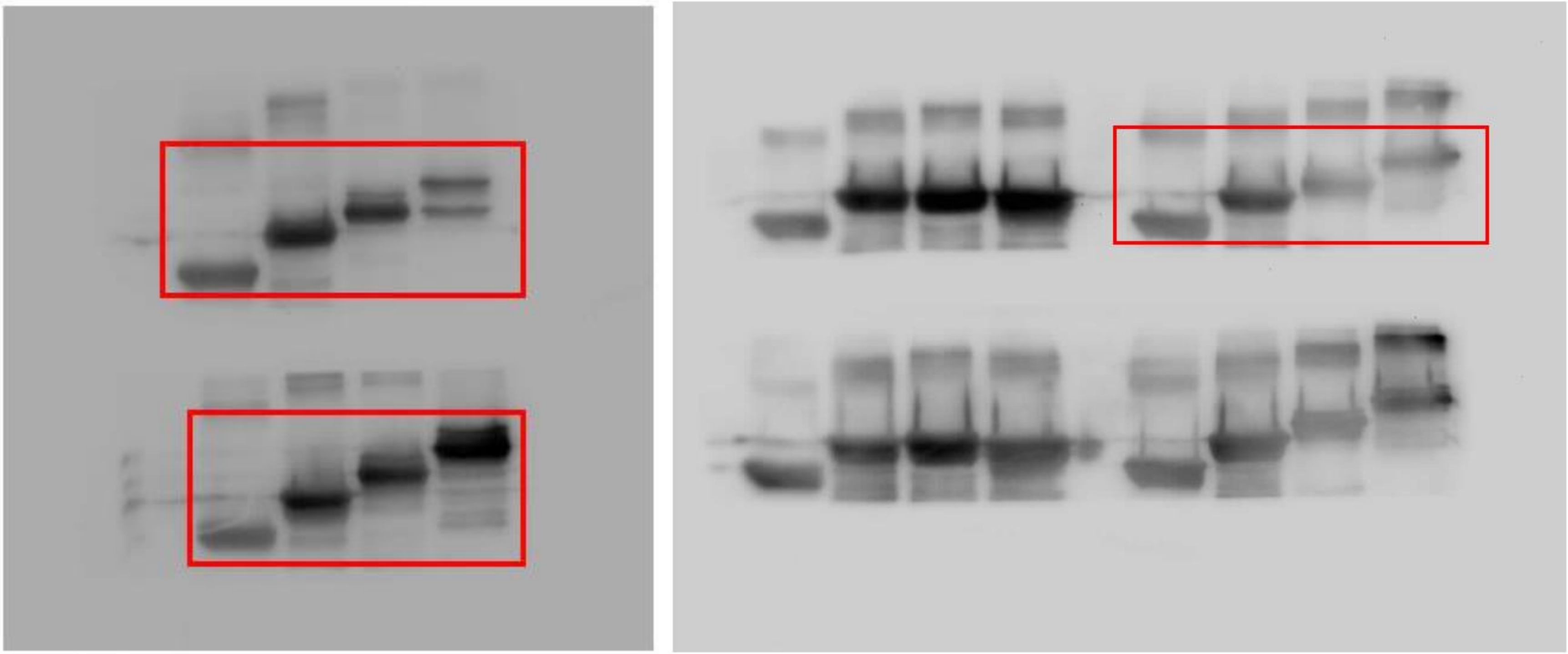

Anti GST

Figure 2B

GABARAP

+ 5µg traps

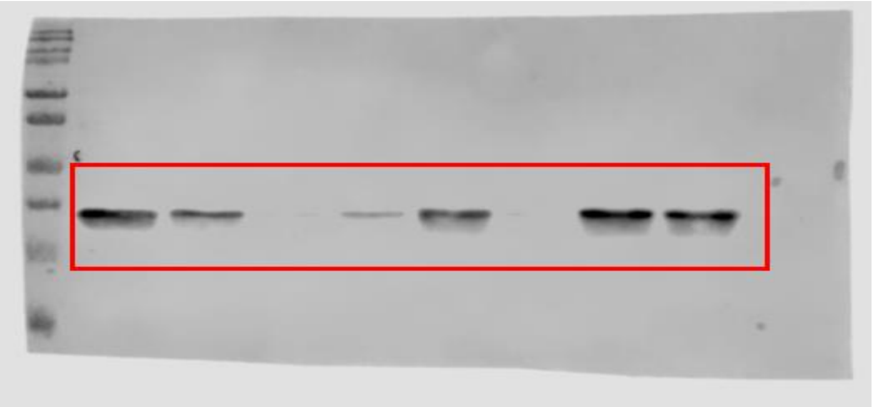

+ 3µg traps

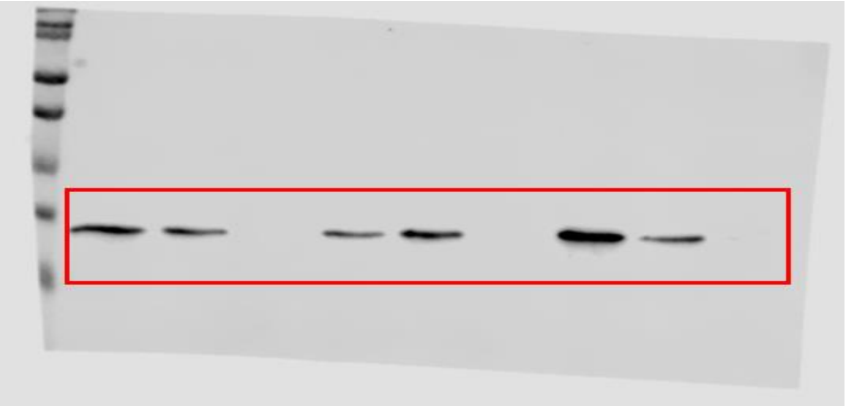

+ 1µg traps

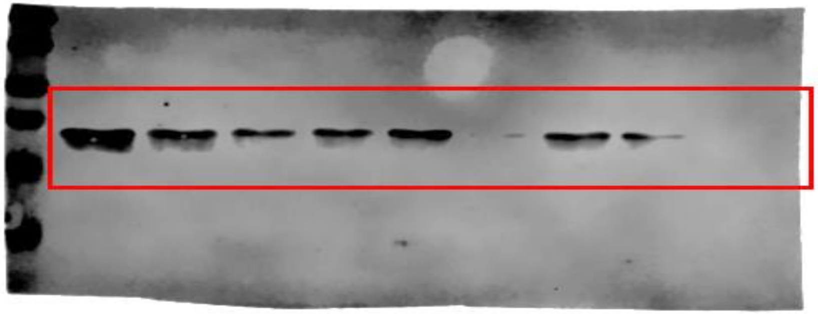

+ 2µg traps

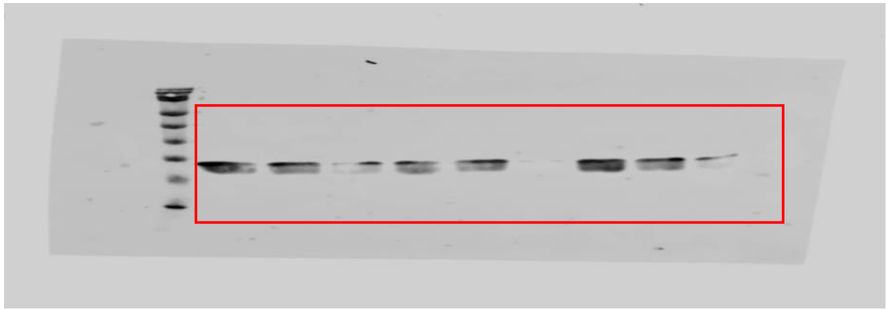

Figure 2B

GABARAP

+ 0,25µg traps

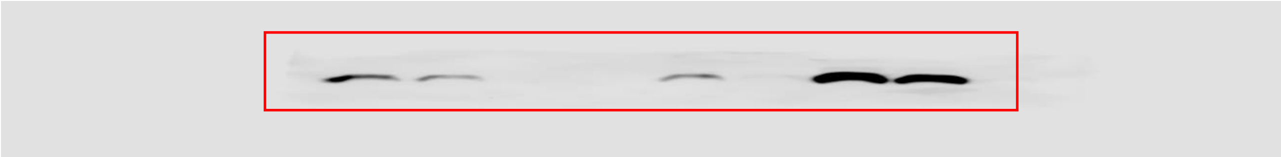

+ 0,125µg traps

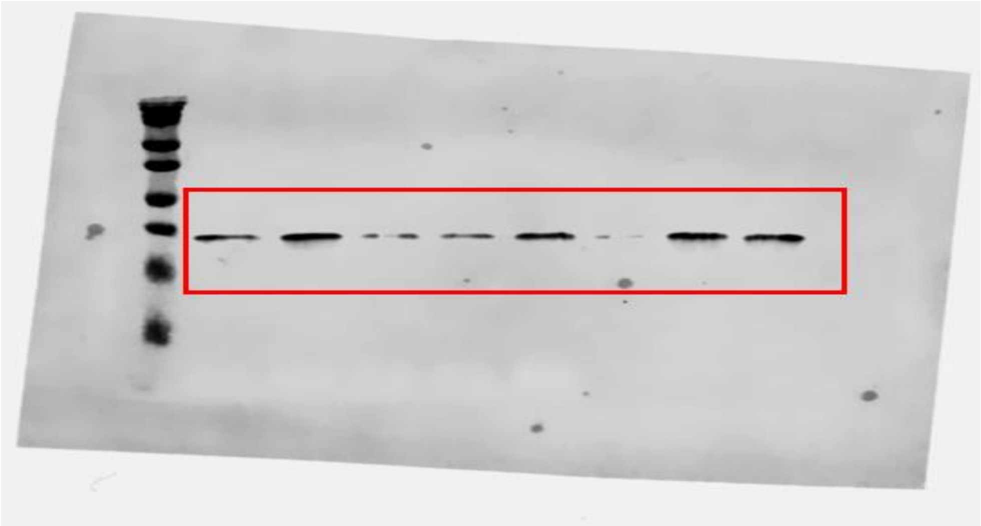

Figure 2C

LC3B

+ 12,5µg traps

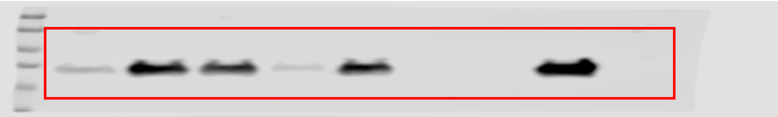

+ 5µg traps

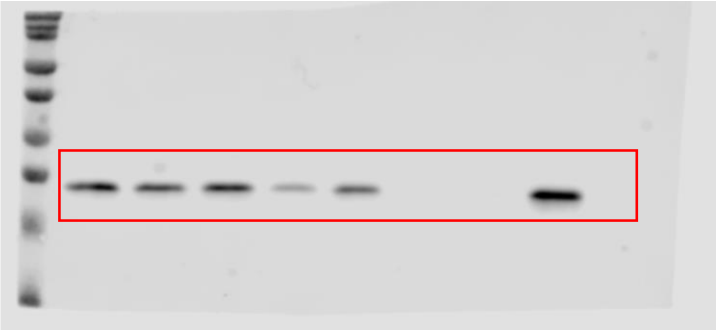

+ 25µg traps

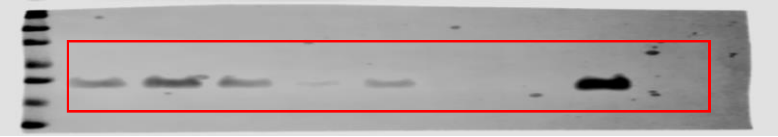

+ 10µg traps

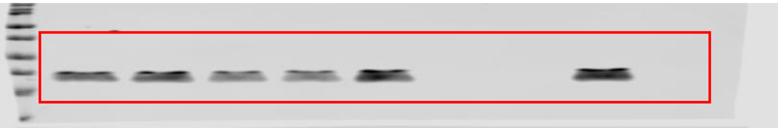

Figure 2C

LC3B

+ 5µg traps

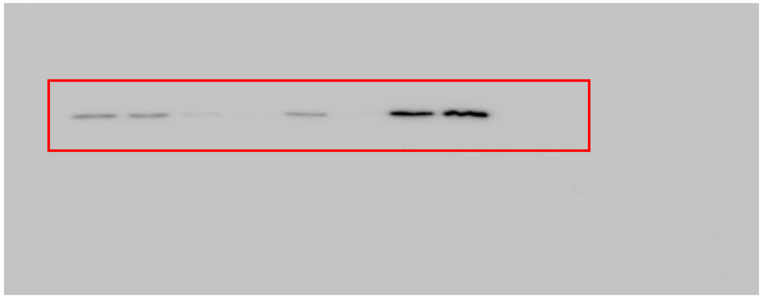

+ 3µg traps

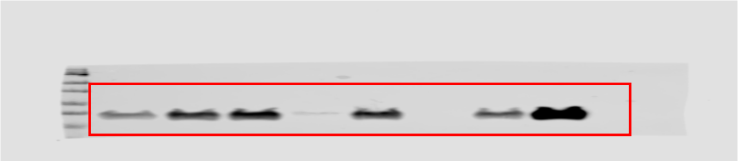

Figure 3A

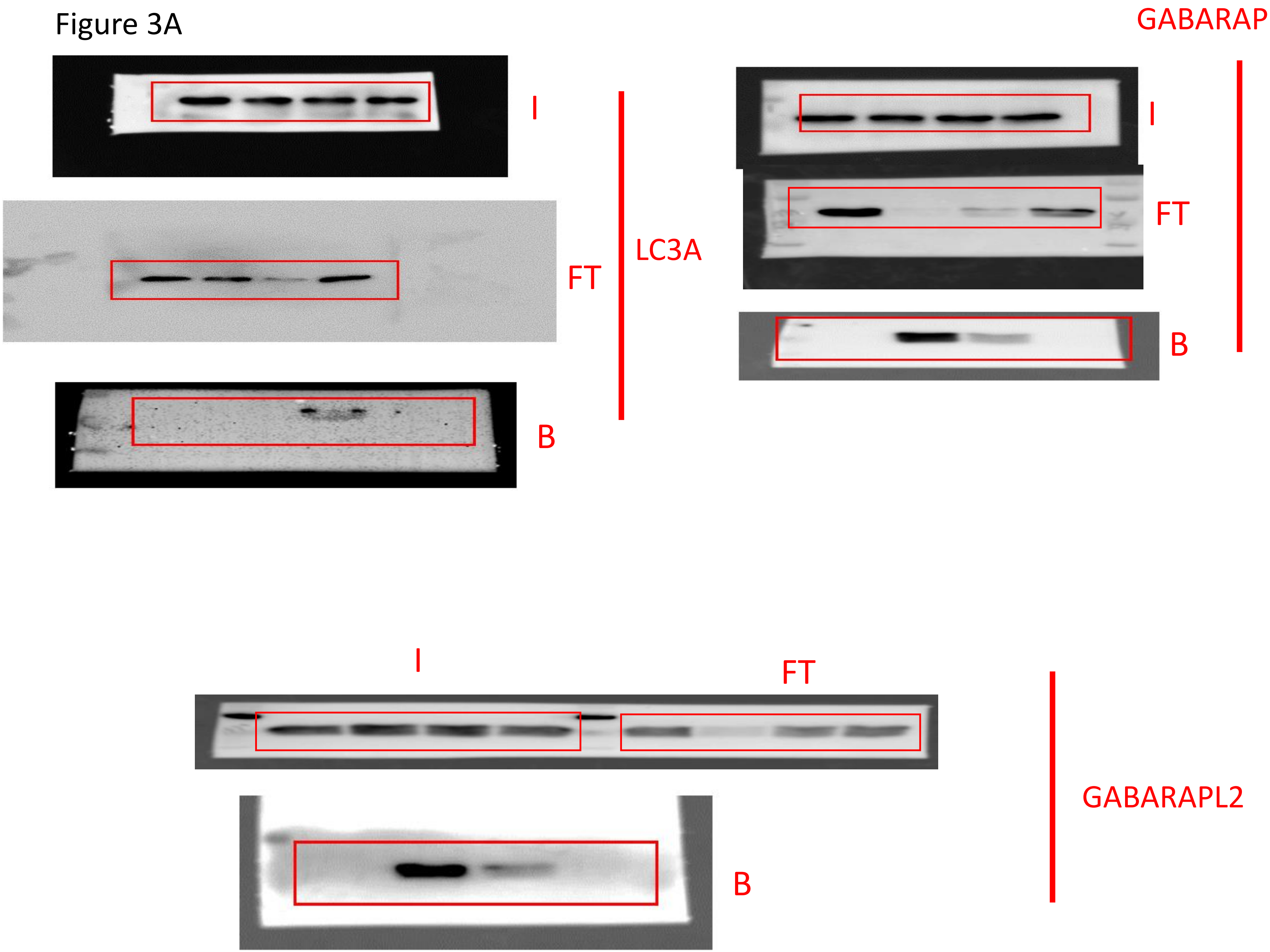

Figure 3A

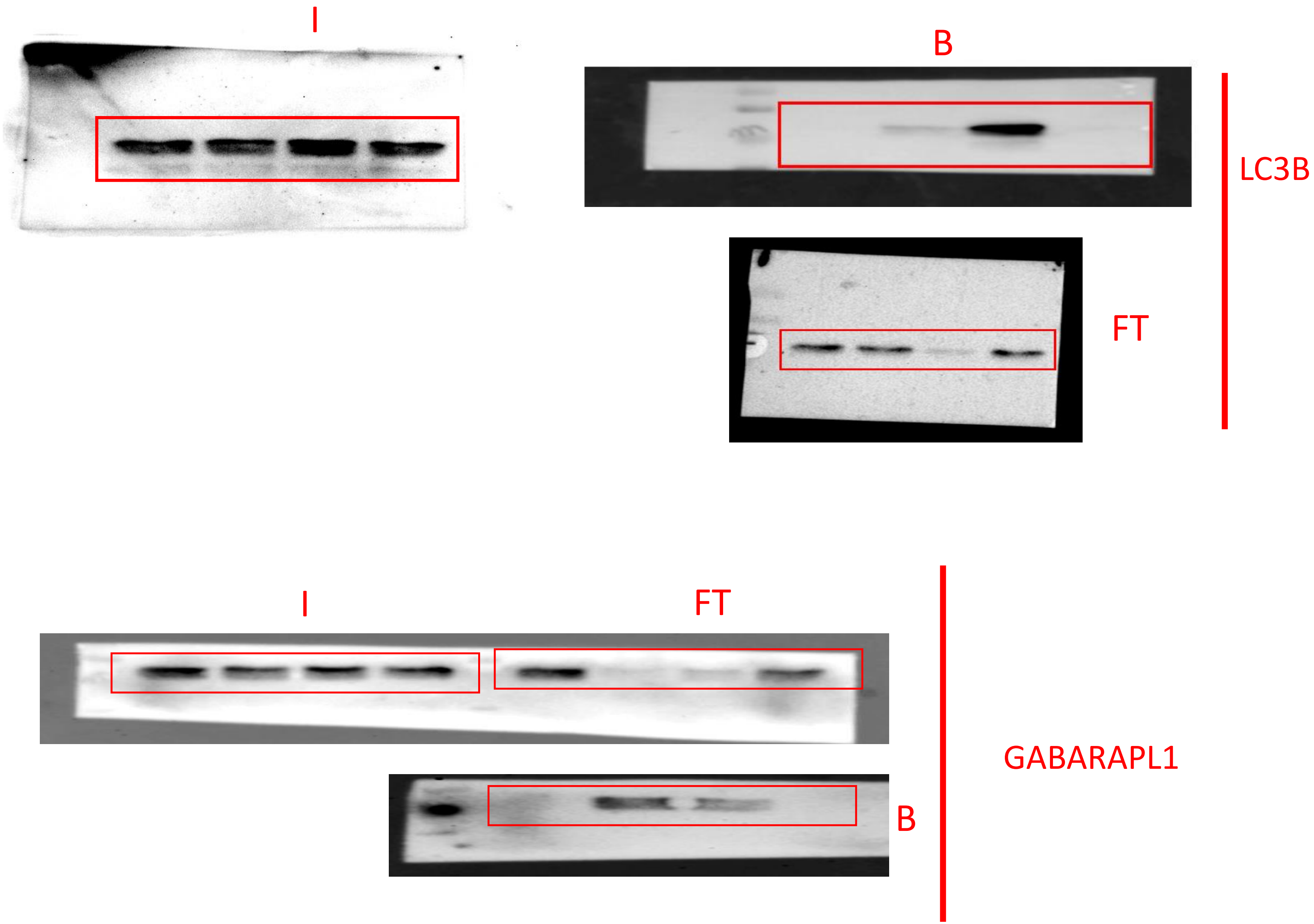

Figure 3B

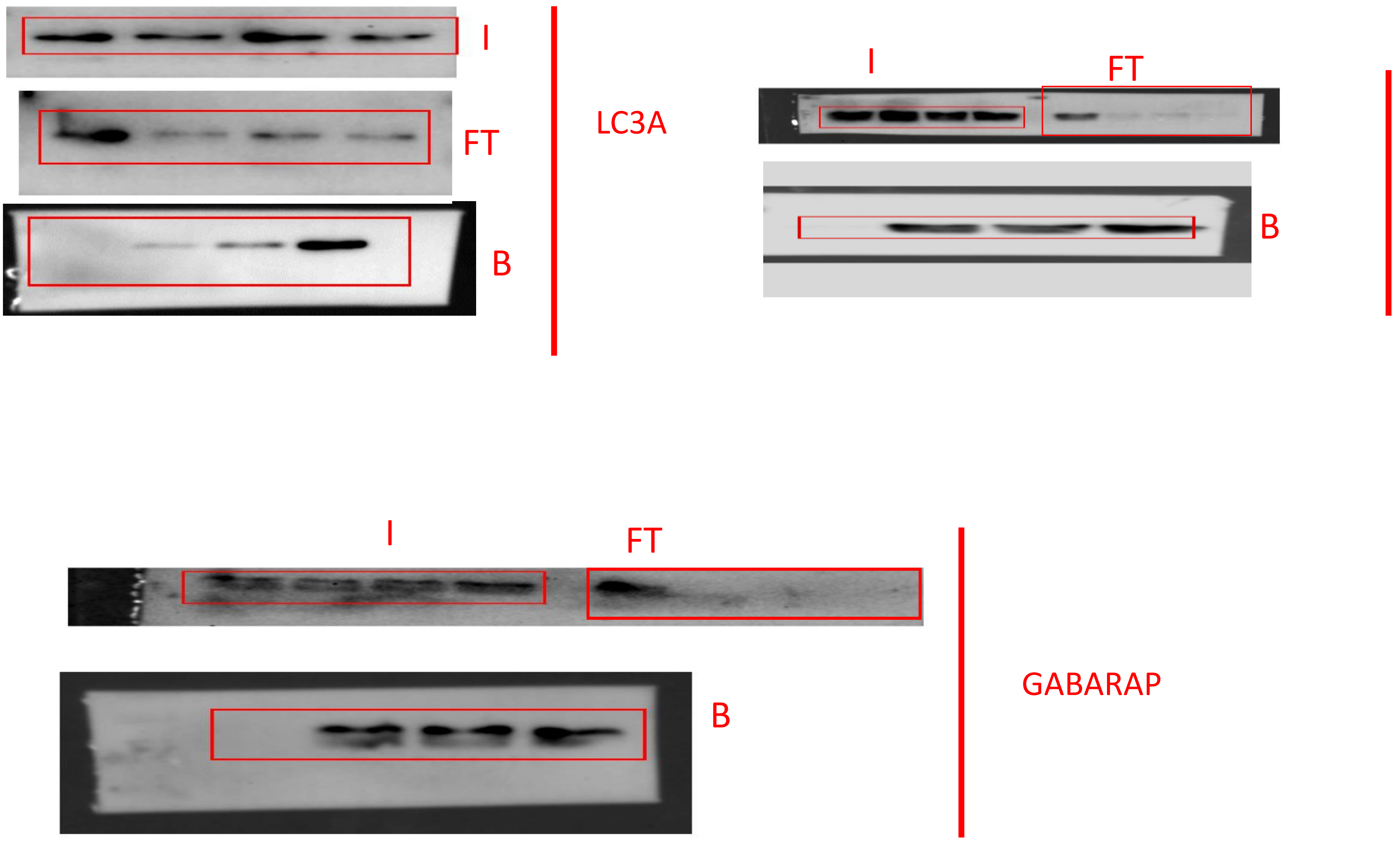

Figure 3B

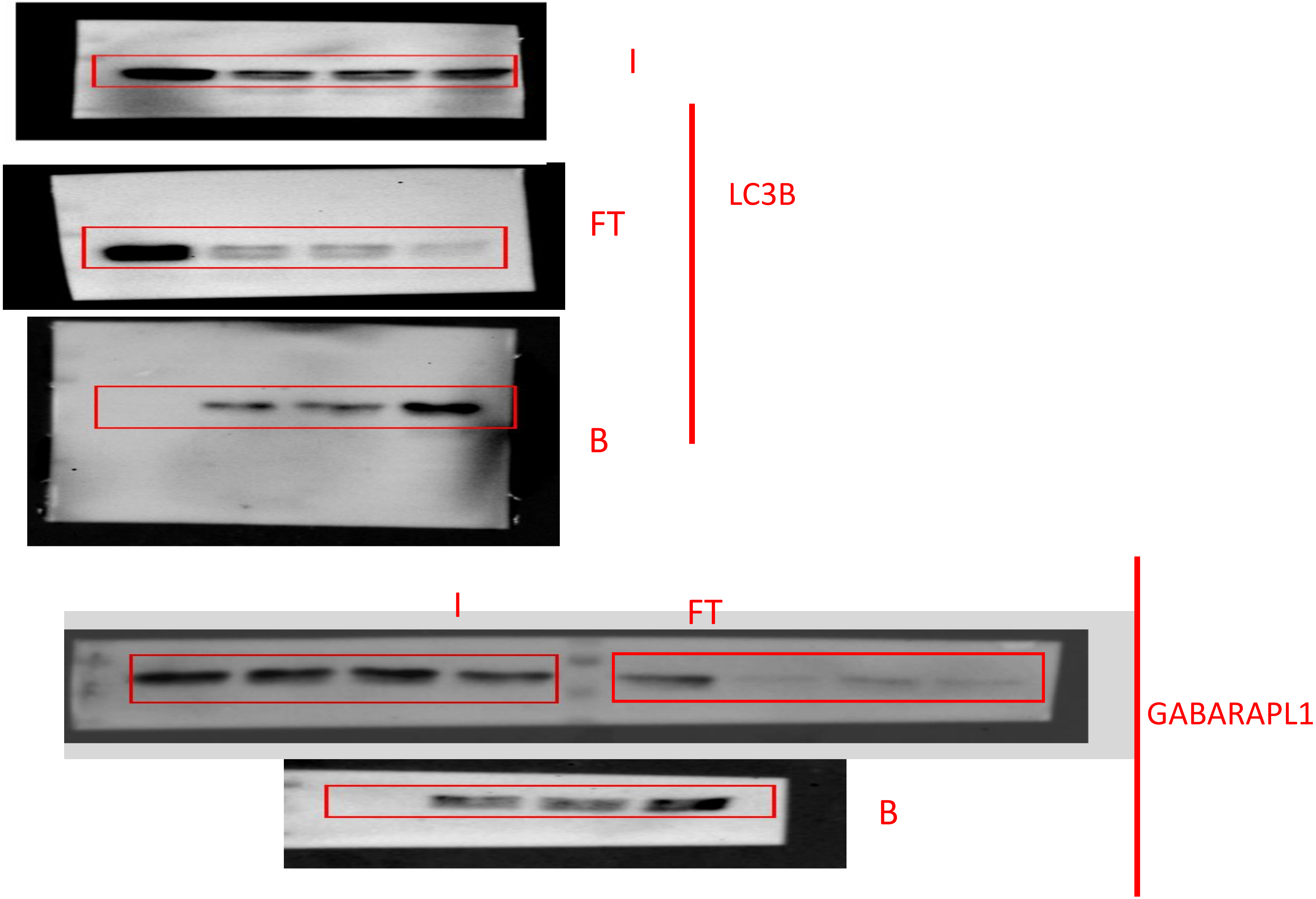

Anti GFP

Figure 4A

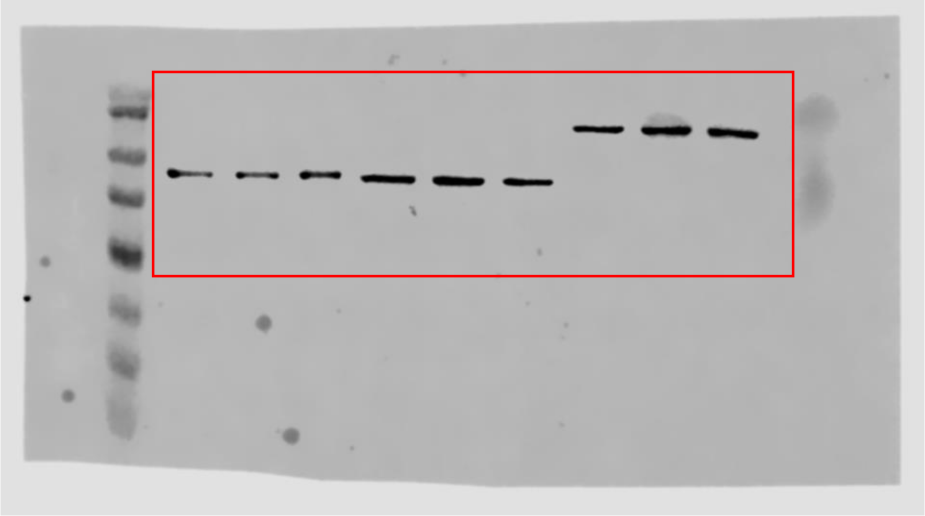

Input

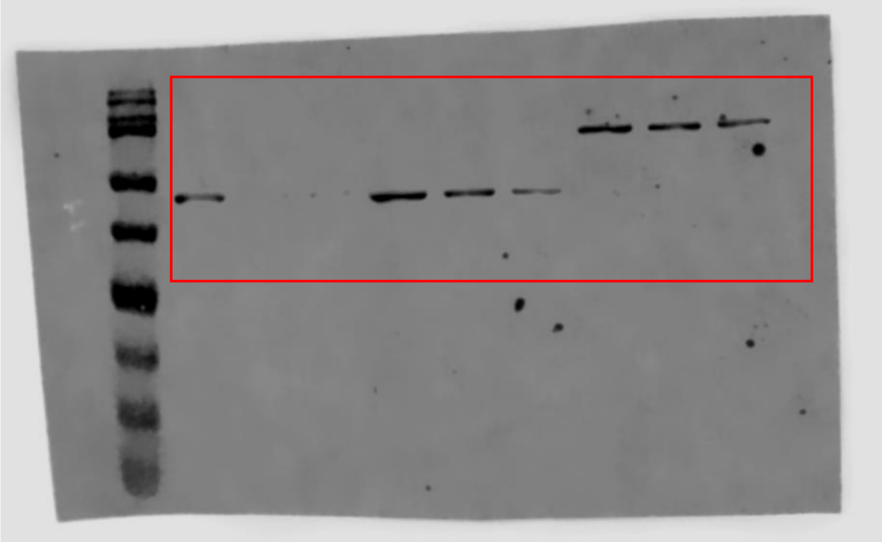

Flowthrough

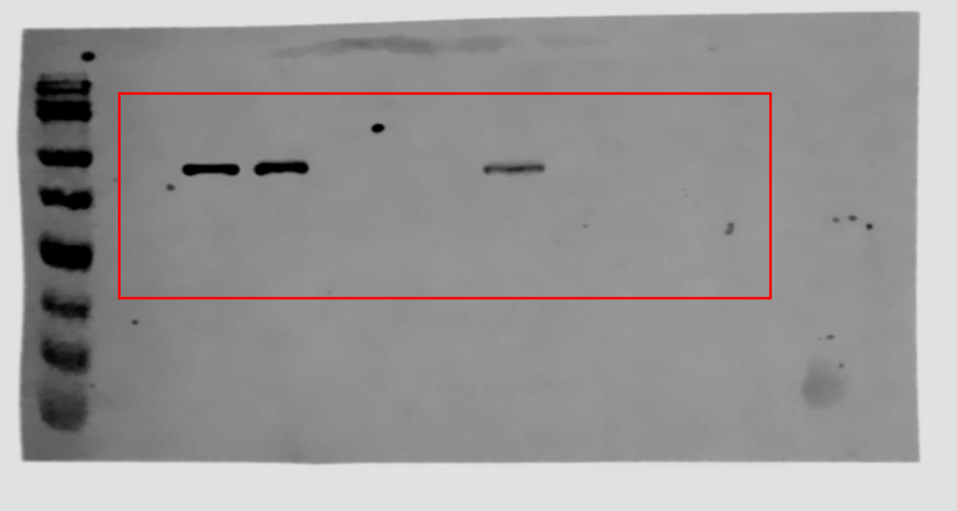

Bound

Figure 4A REPLICAT 2

Anti GFP

Input

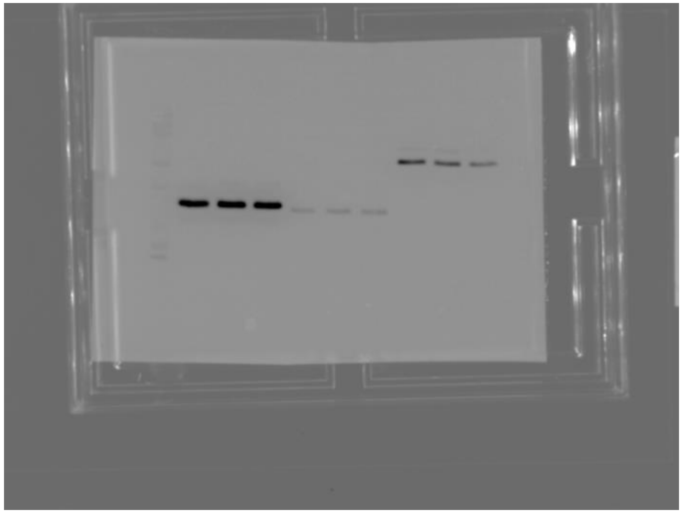

Bound

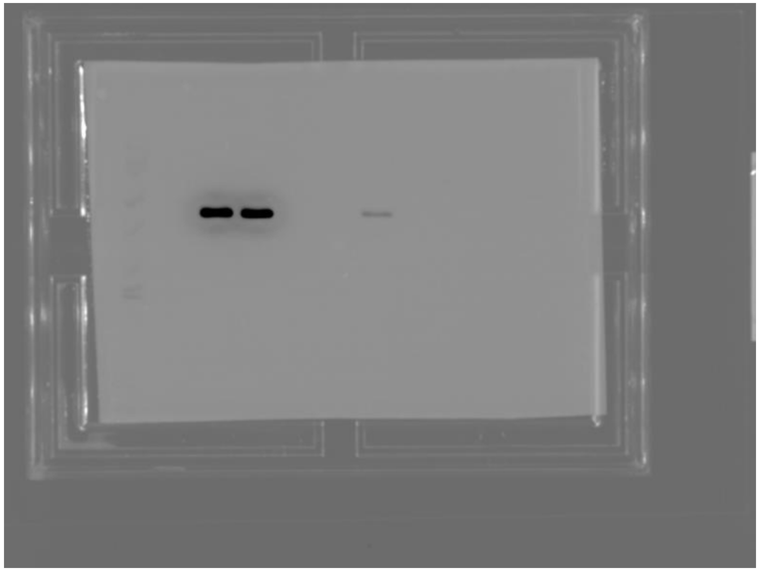

Anti GFP

Figure 4A REPLICAT 3

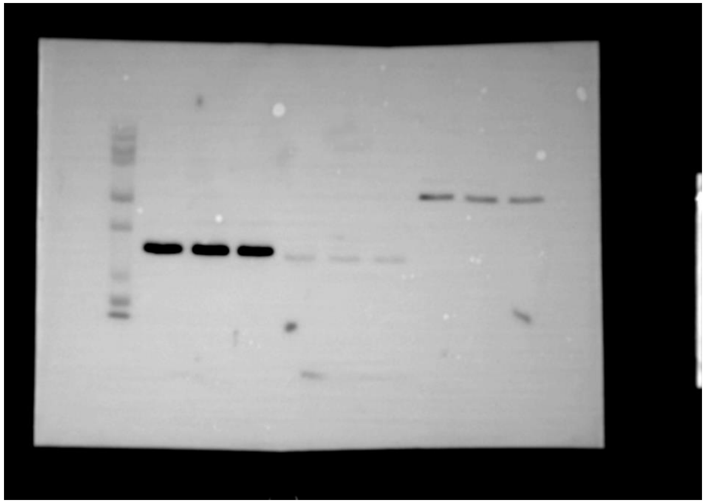

Input

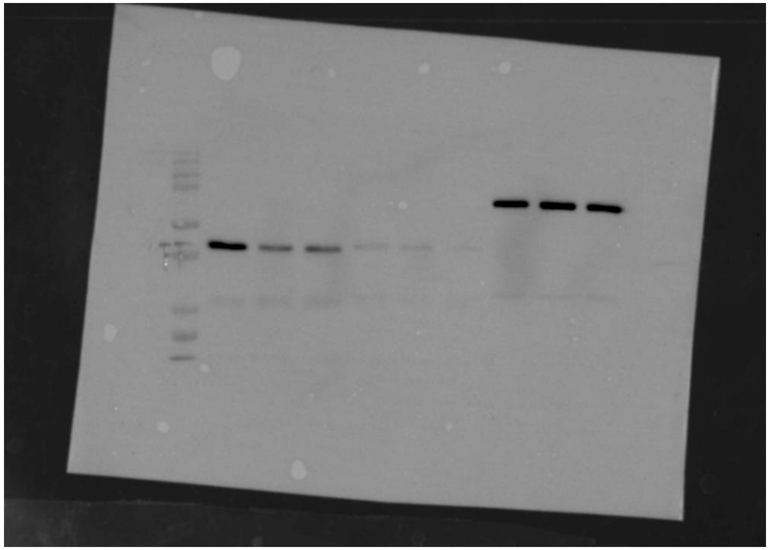

Flowthrough

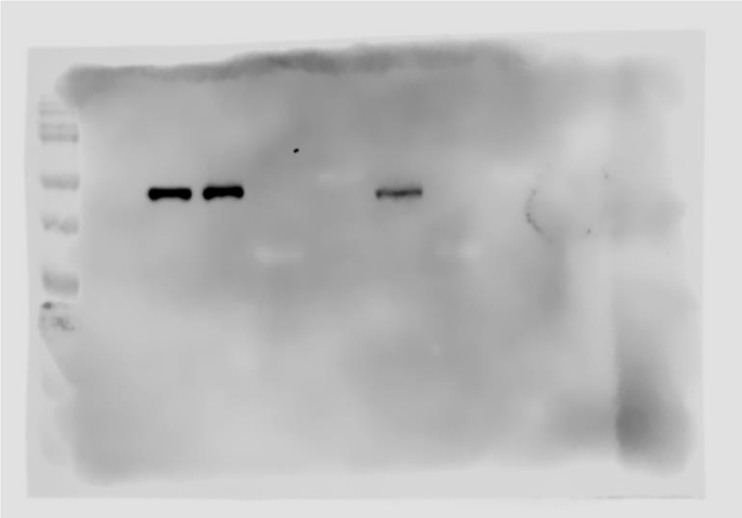

Bound

Anti GFP

Figure 4B

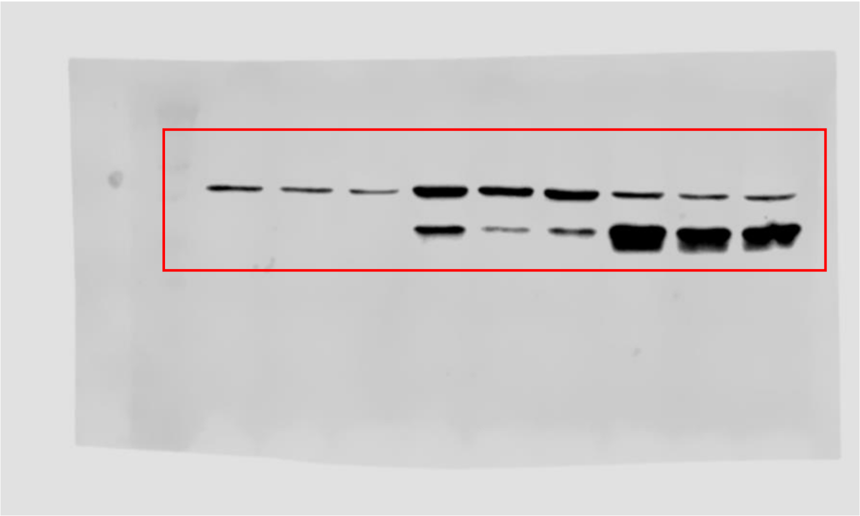

Input

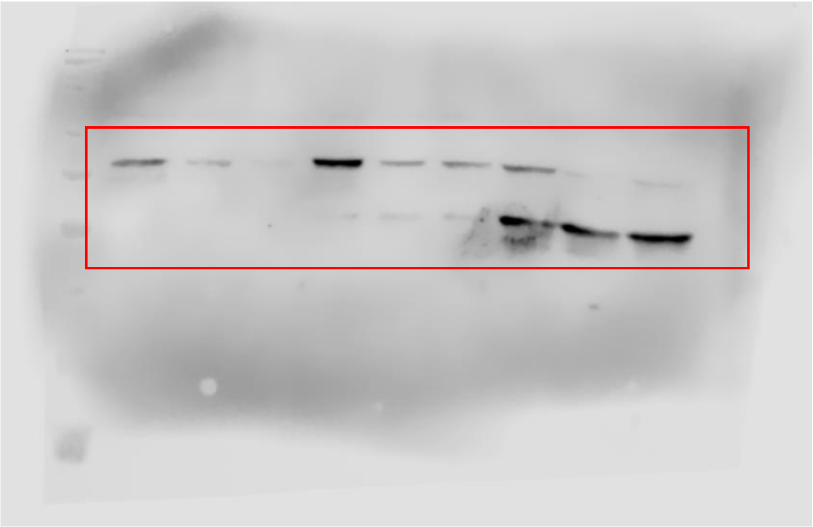

Flowthrough

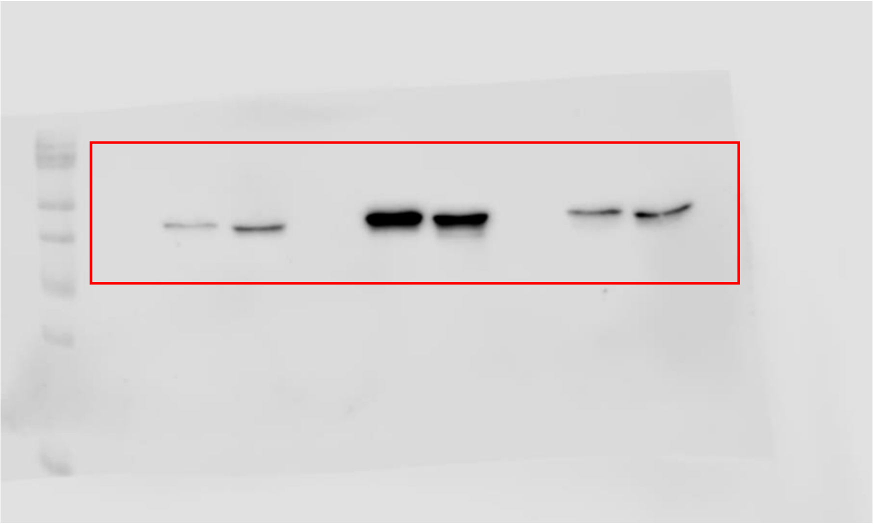

Bound

Figure 4B REPLICAT 2

Anti GFP

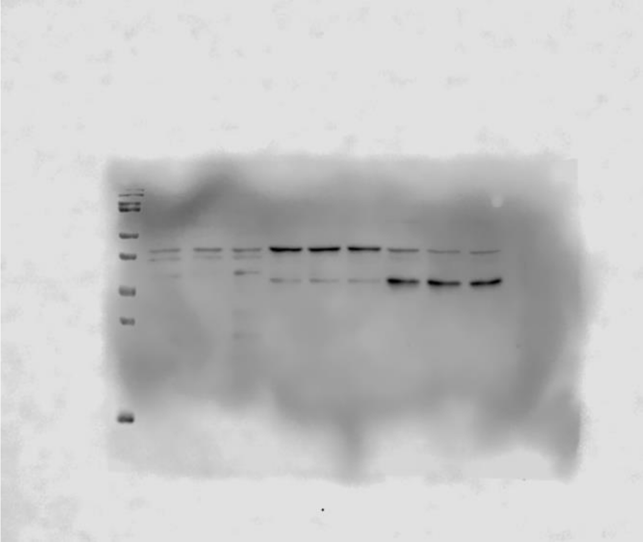

Input

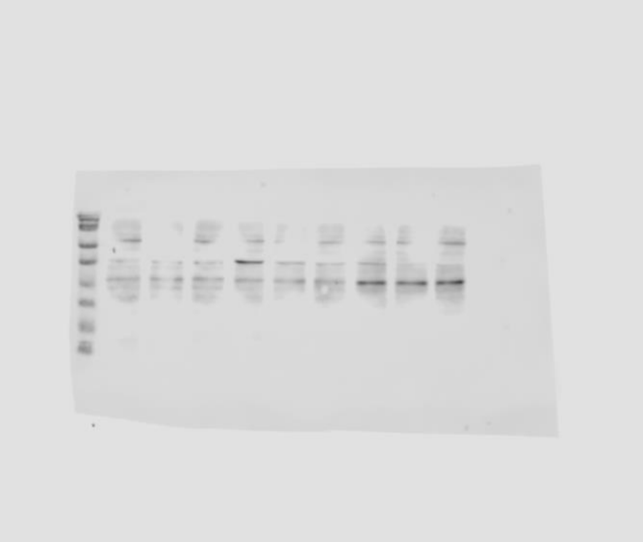

Flowthrough

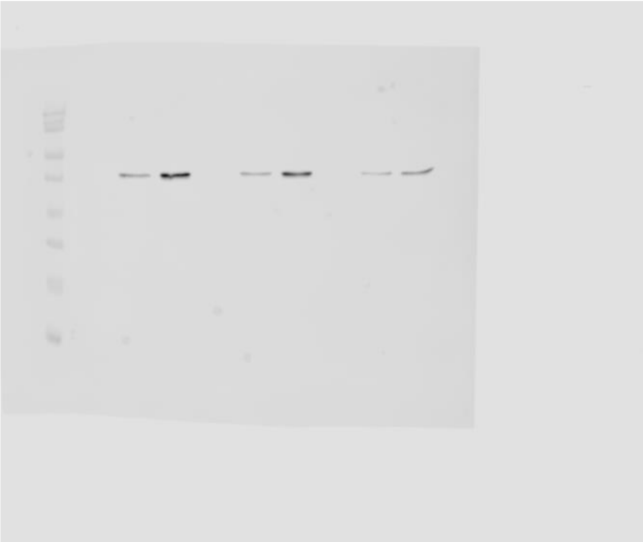

Bound

Figure 4C

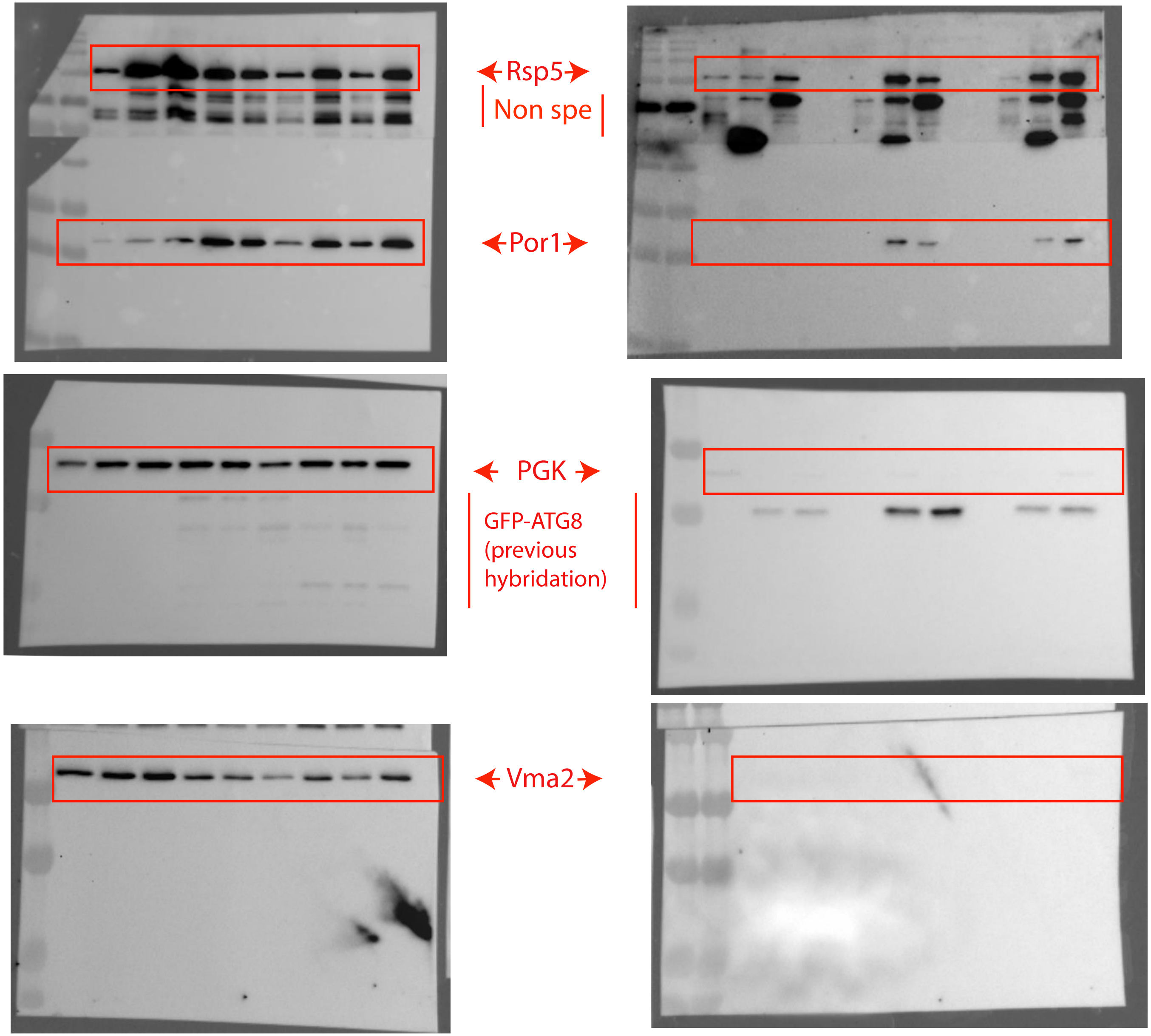

Figure 5A

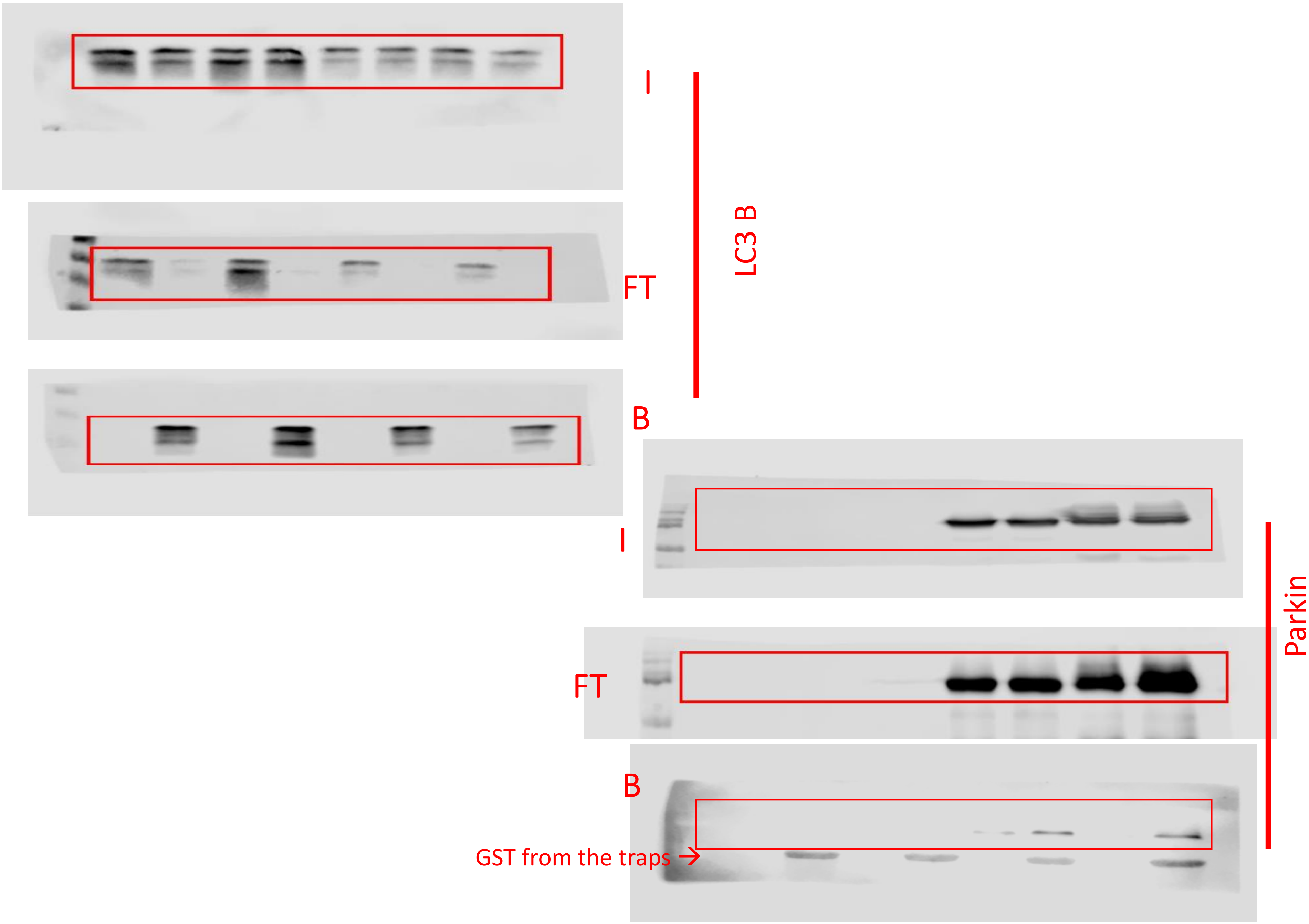

Figure 5A

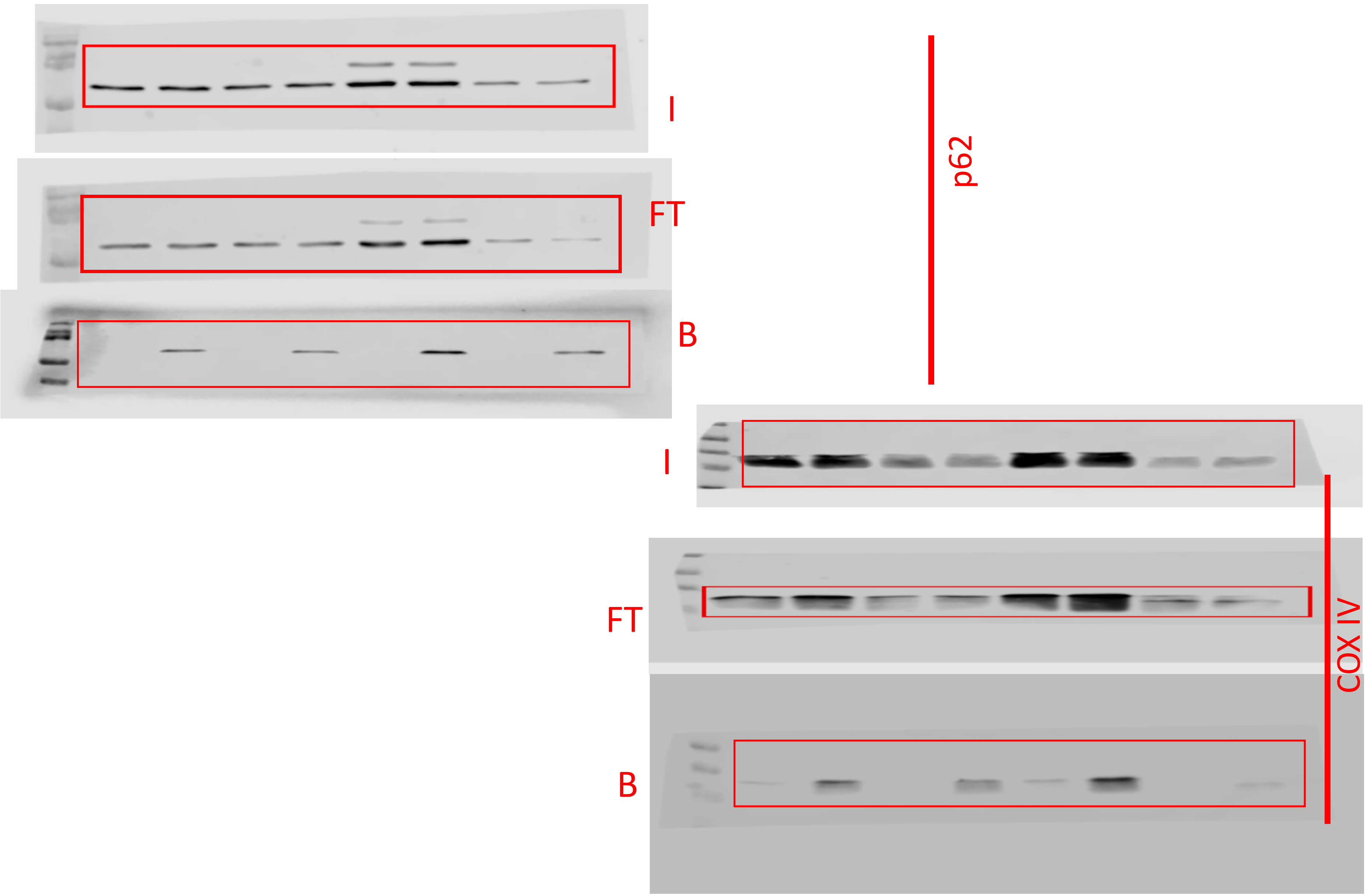

Figure 5A REPLICAT 2

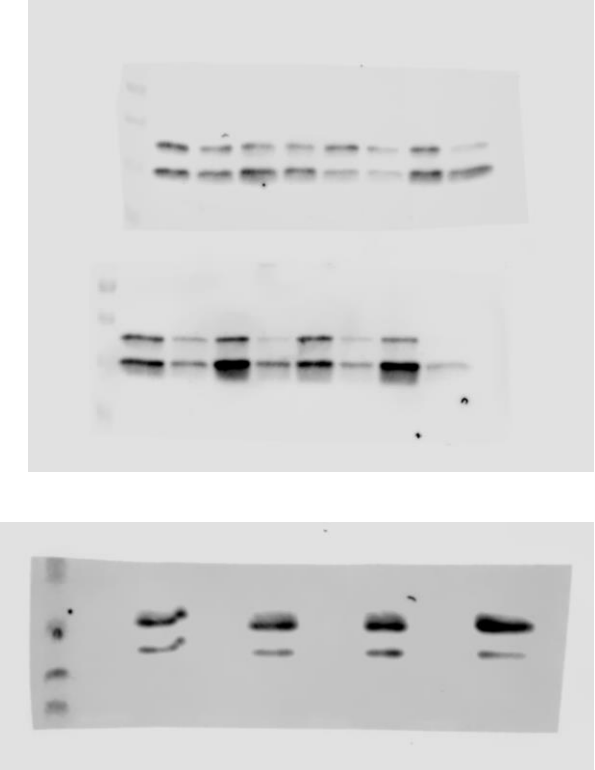

I

FT

B

LC3 B

p62

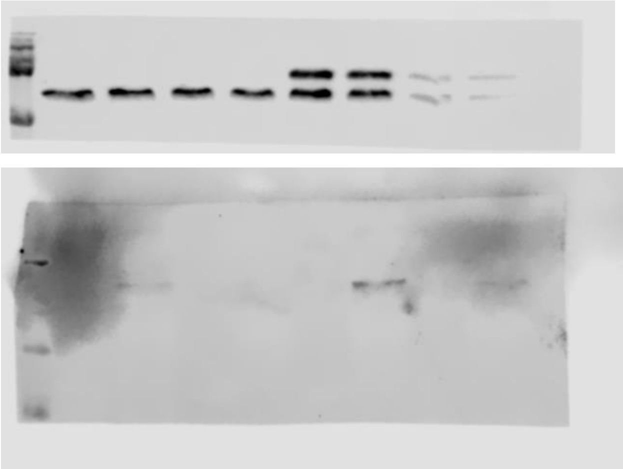

I

B

Parkin

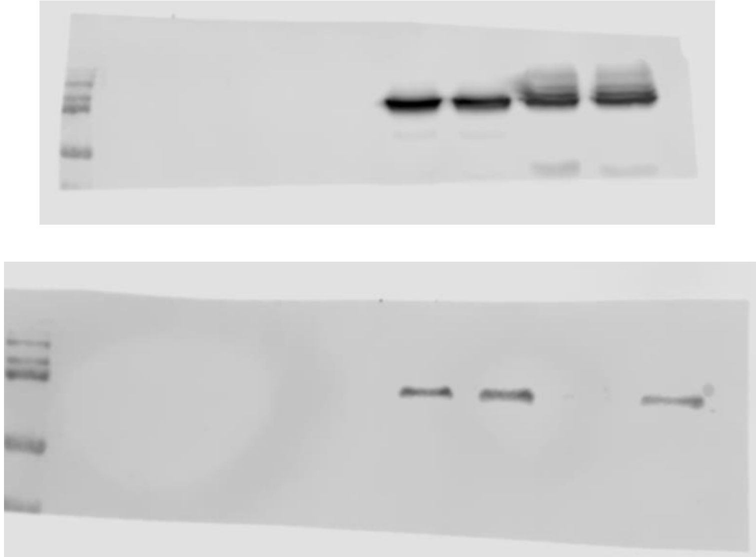

I

B

Figure 5B

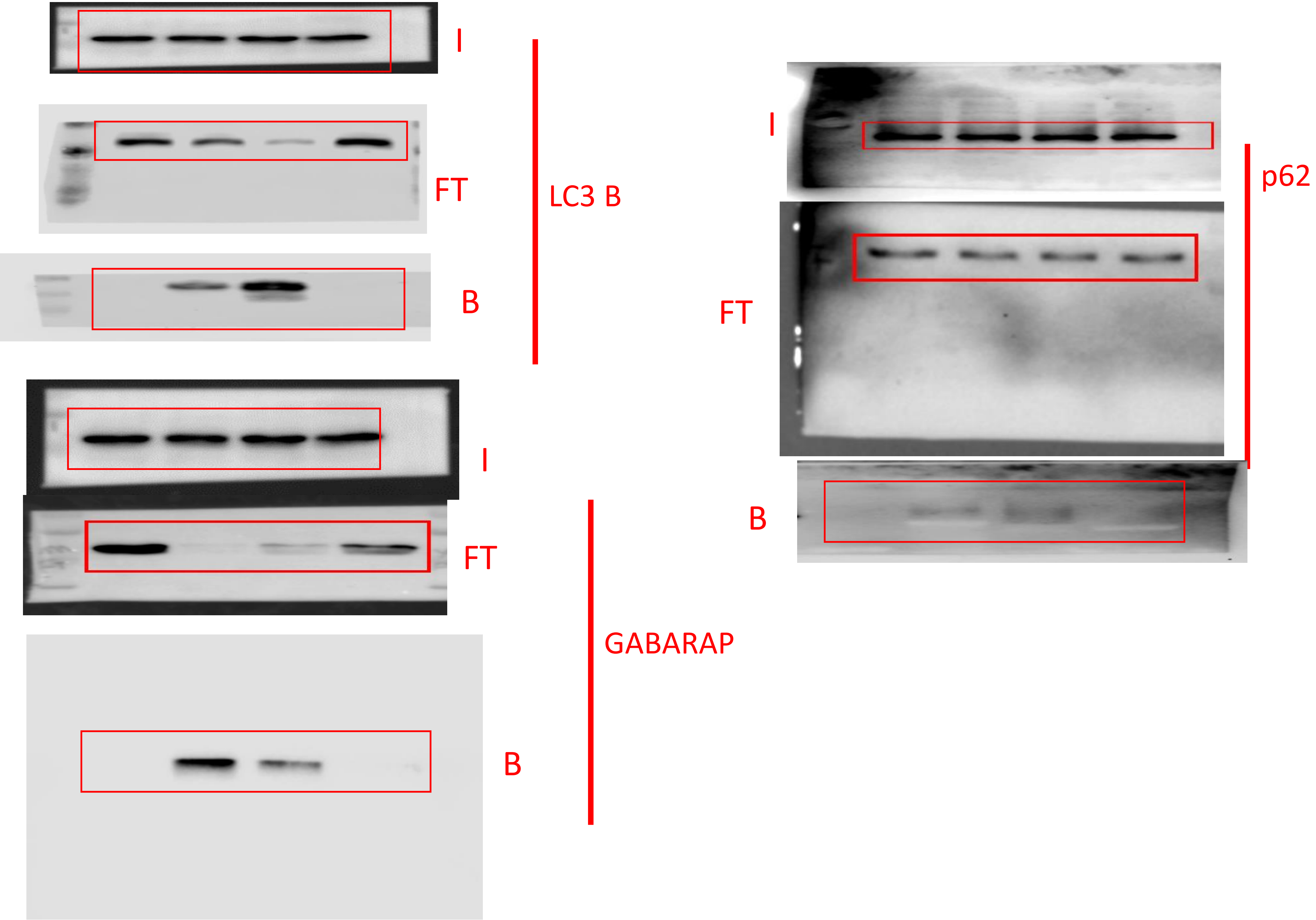

Figure 5B

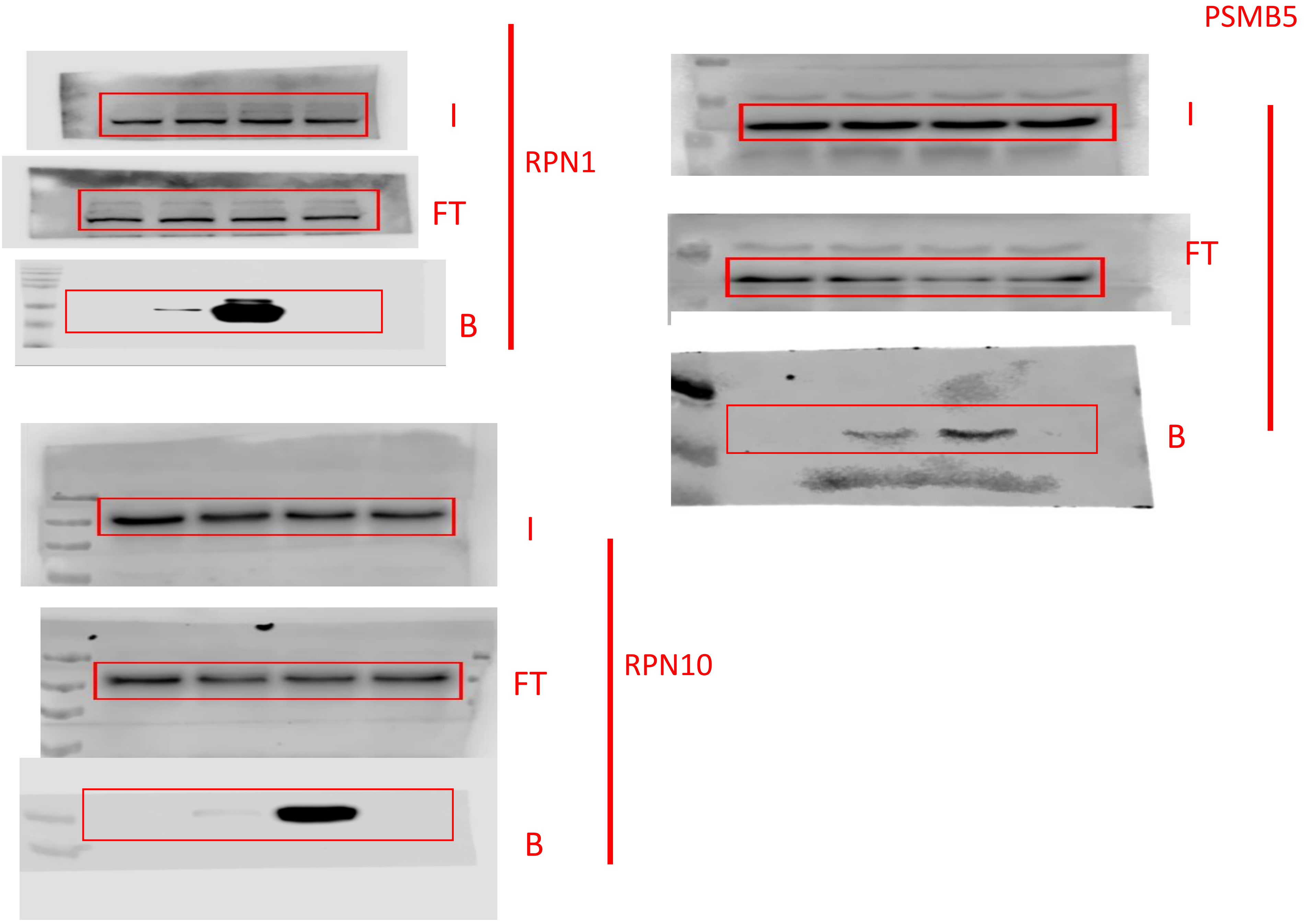

Figure 5B REPLICAT 2

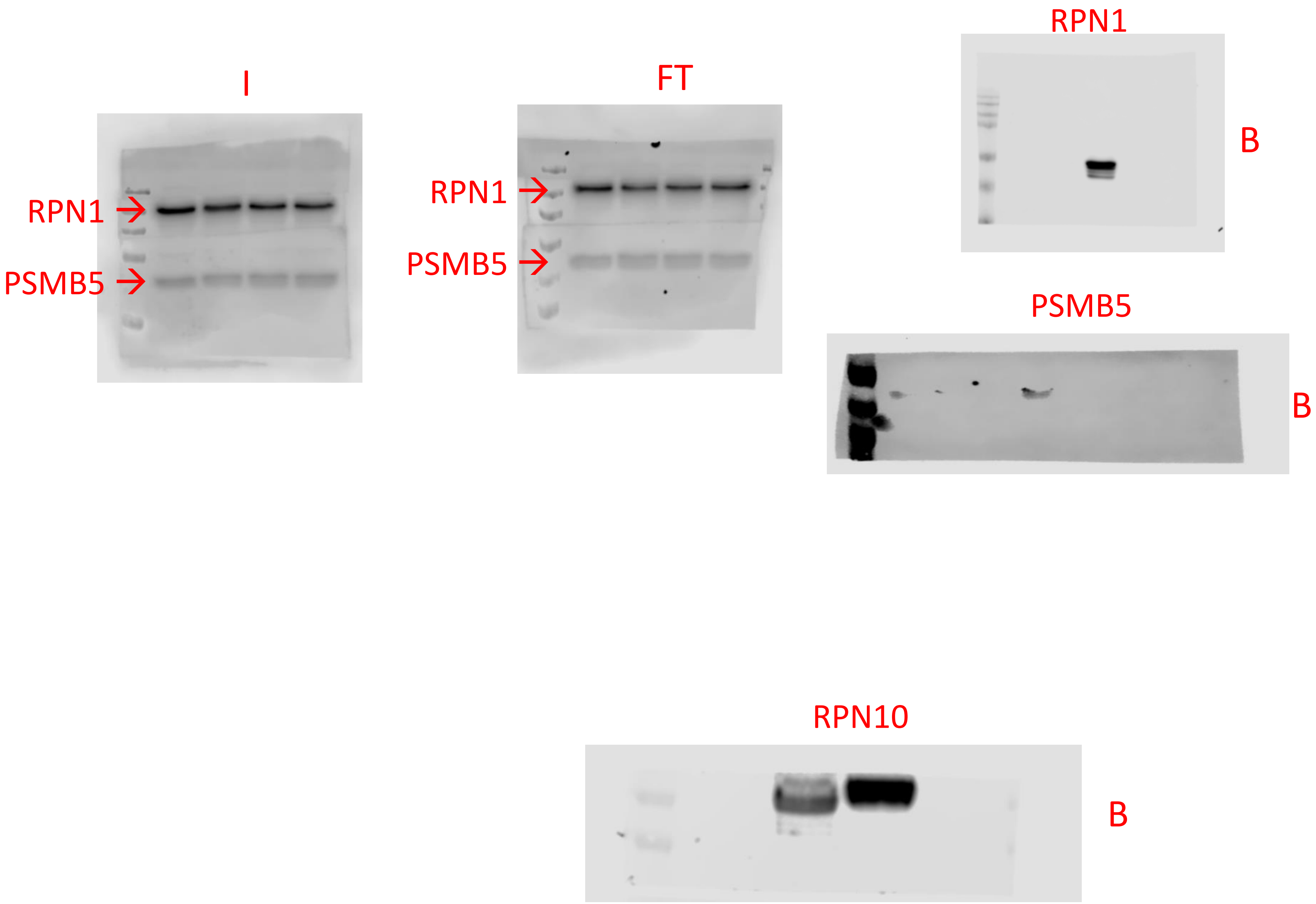

Supplementary Figure 2

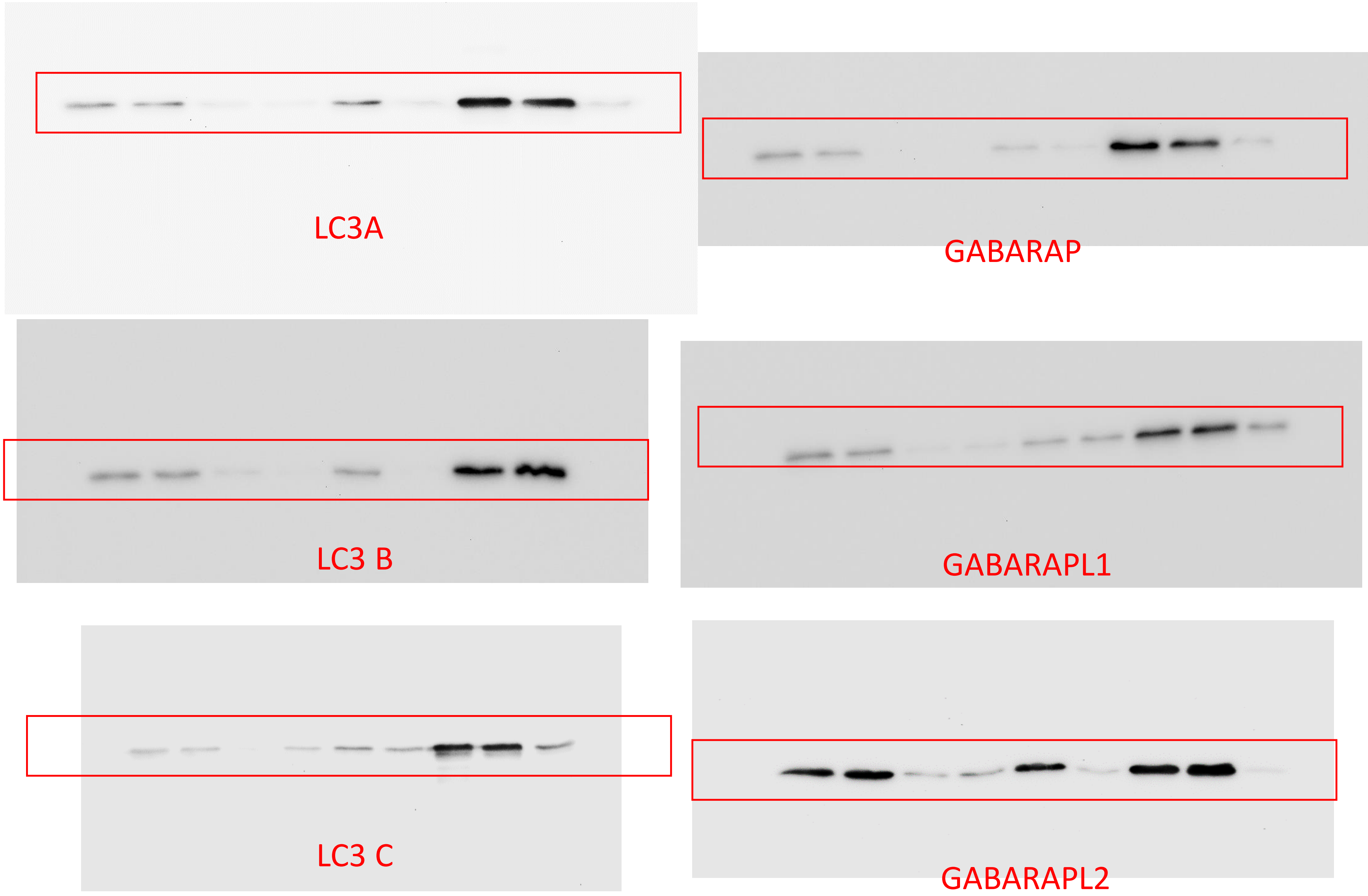

Supplementary Figure 3

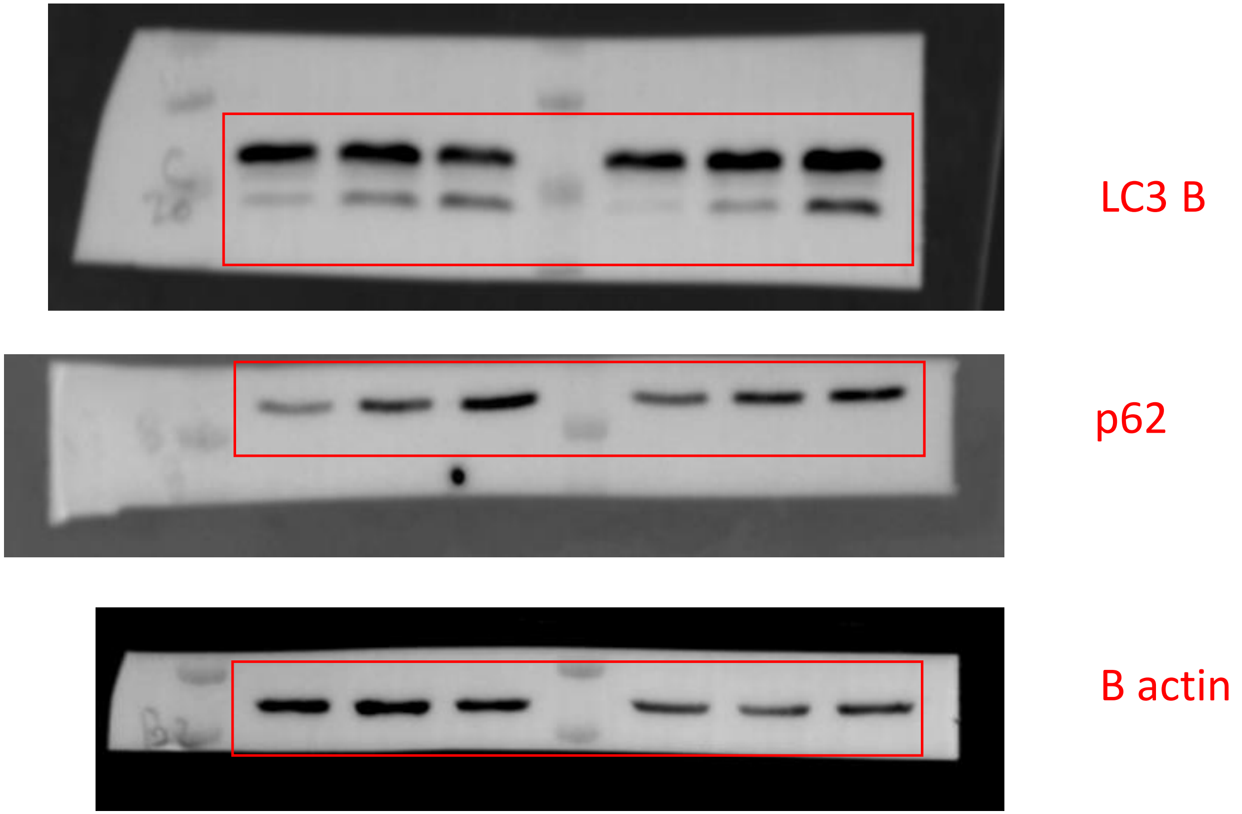

Supplementary Figure 4

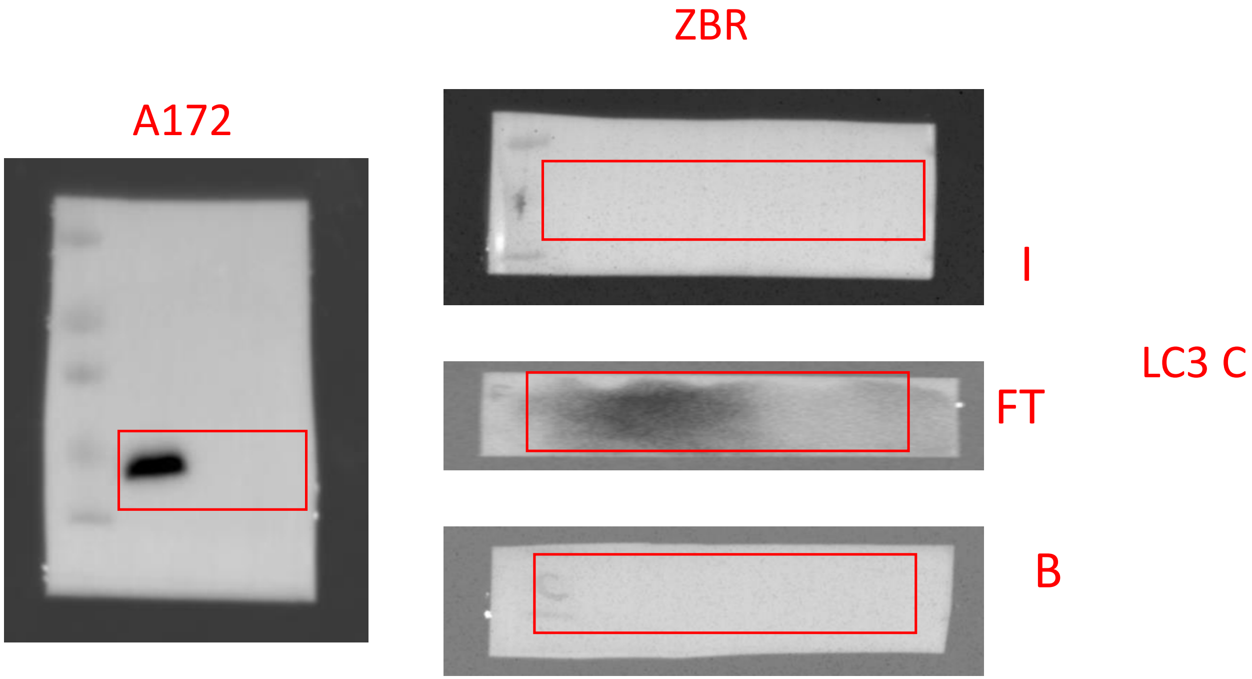

Supplementary Figure 5

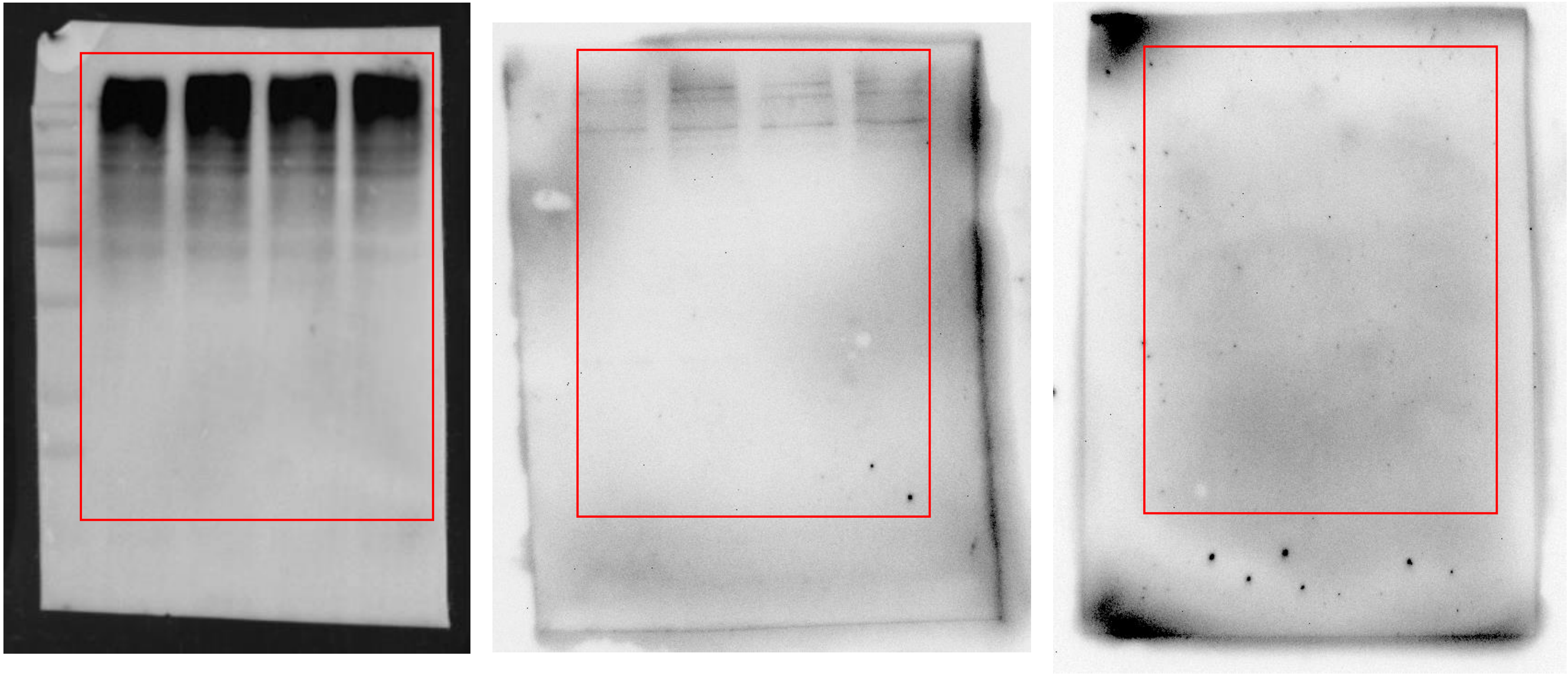

Ubiquitin
