# Supplementary material for: Exploring selective autophagy events in multiple biologic models using LC3-interacting regions (LIR)-based molecular traps
Source: Sci Rep. 2022 May 10;12:7652. doi: 10.1038/s41598-022-11417-z (PMC9090809; doi:10.1038/s41598-022-11417-z)
Supplement: Supplementary file 2 — Supplementary Information 2. [file 41598_2022_11417_MOESM2_ESM.docx]

**Supplemental information**

**Figures and legends**

**
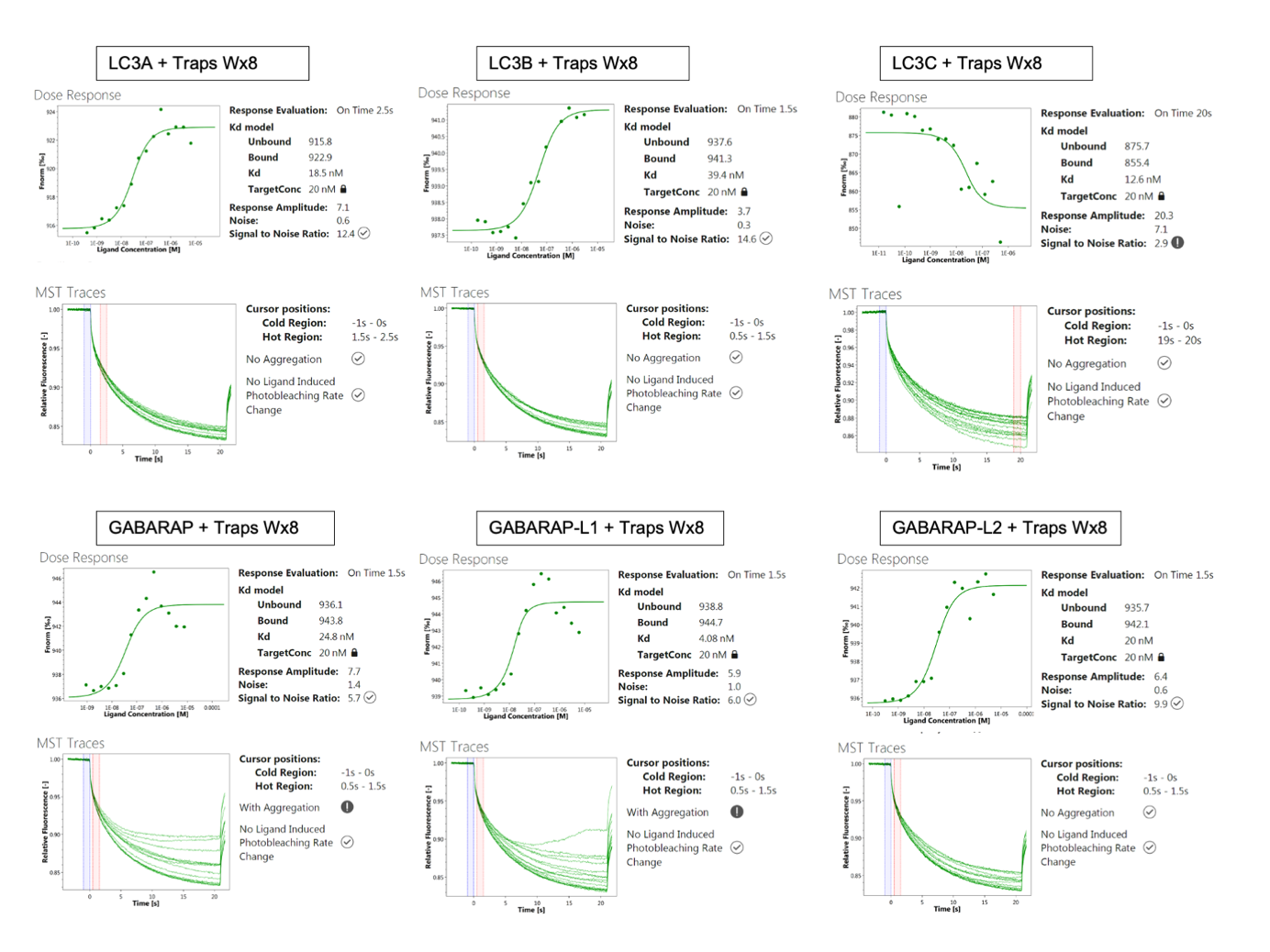
**

**Supplementary Figure 1. Analysis of LC3-traps affinity by MST.**  Relative Fluorescence of the 16 binding reactions was measured in a time-dependent manner. Binding reactions were set with a constant concentration (20nM) of Trap Wx8 and with LC3A, LC3B, LC3C, GABARAP, GABARAPL1 or GABARAPL2 diluted from 3µM to 91nM. Kd values for all traps-LC3/GABARAP protein interactions were calculated by Nanotemper software. Values are displayed in Figure 2A.

**
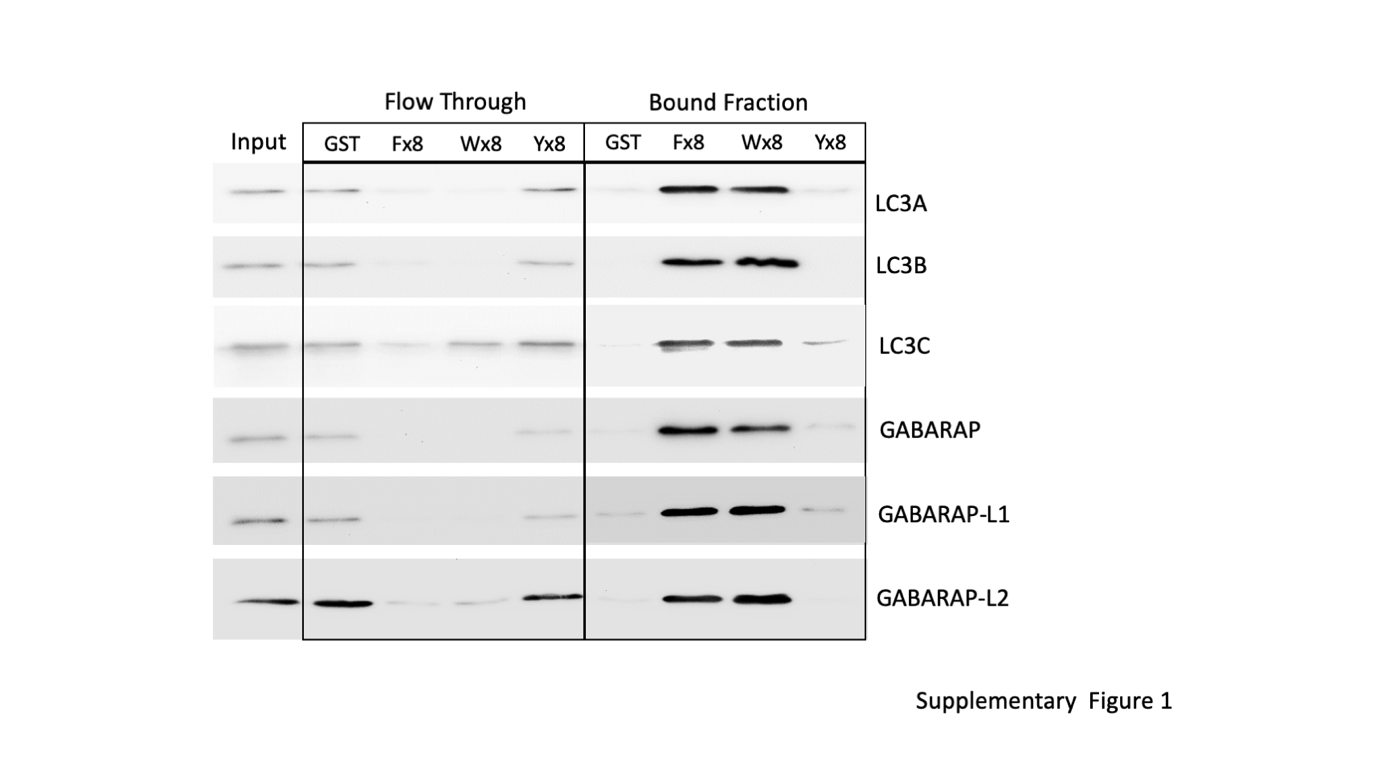
**

**Supplementary Figure 2. Capture of recombinant LC3/GABARAP proteins by LC3-traps with 8 tandem repeats.** Five micrograms of LC3-traps Fx8, Wx8, Yx8 were used to capture a fixed amount (1μg) of each recombinant LC3/GABARAP protein. After overnight incubation, input, flow-through and bound fractions were analyzed by Western blot with specific antibodies.


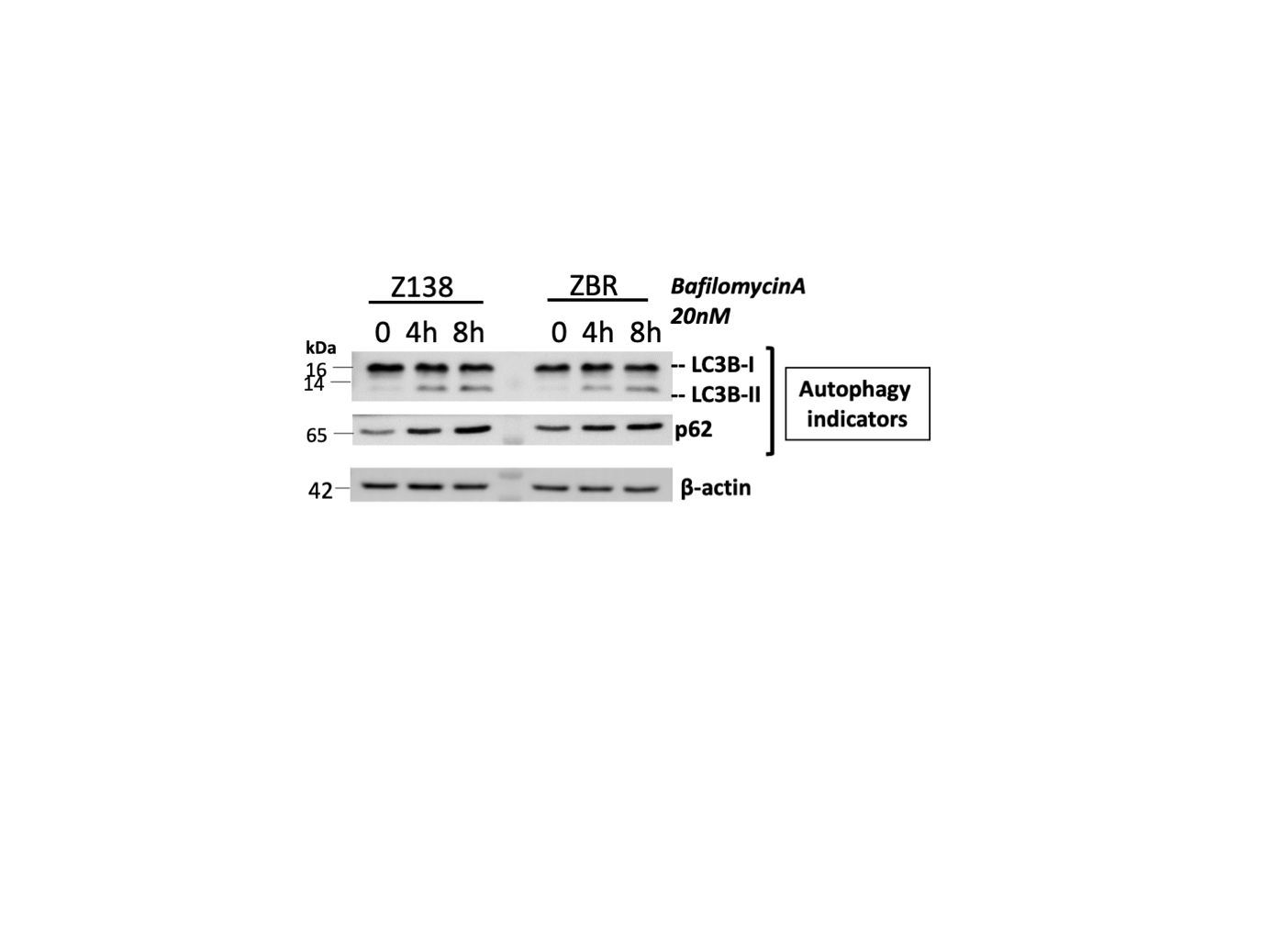


**Supplementary Figure 3. Autophagy is observed after 4 or 8h treatment of Z138 and ZBR cells with bafilomycin A (20nM).** Cell extracts from BTZ sensitive (Z138) and resistant (ZBR) cells treated with Bafilomycin A were used to analyze autophagy. Total cell extracts were analyzed by Western blot to detect LC3B and p62. β-actin was used as a charge control.

**
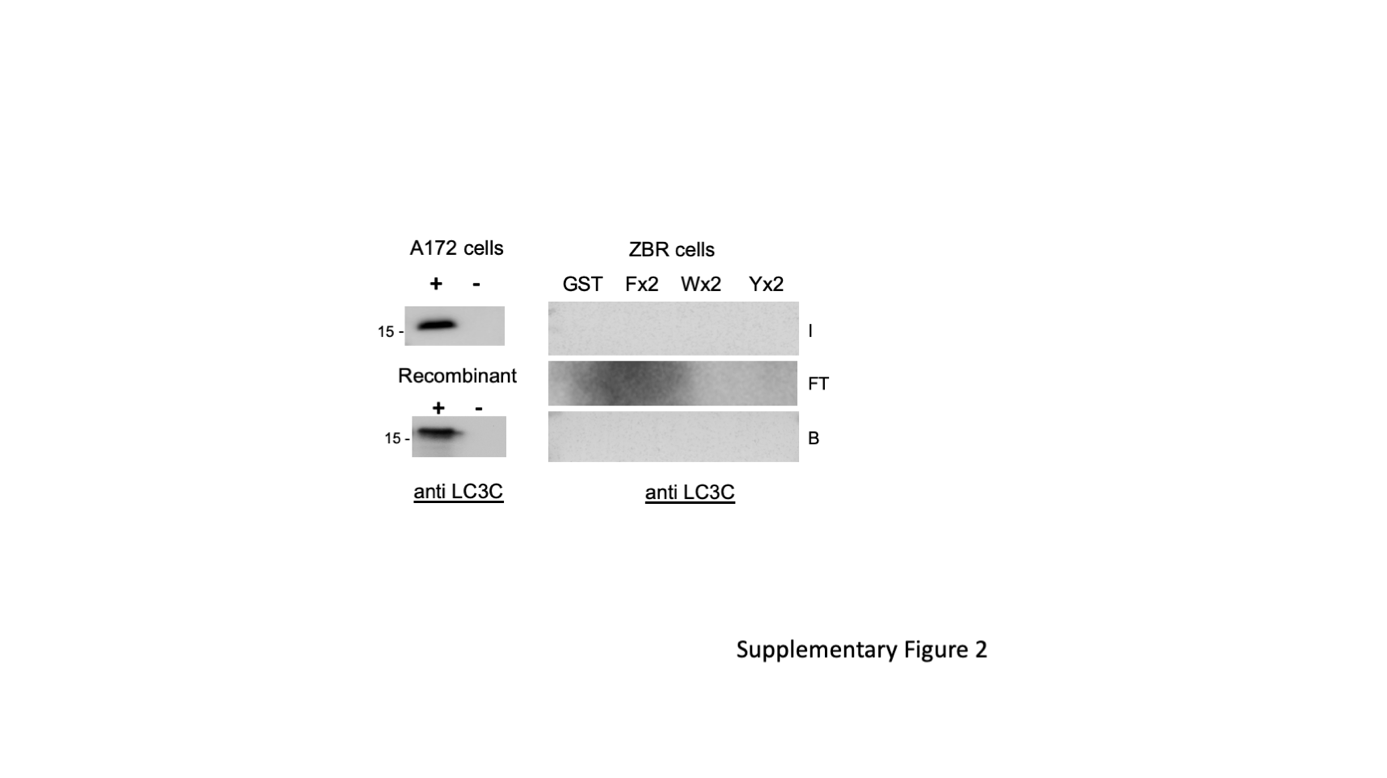
**

**Supplementary Figure 4. LC3C is not detectable in ZBR cells treated with bafilomycin A (8 hours at 20nM).** Cell extracts from ZBR cells treated with Bafilomycin A were used to capture LC3C with the indicated LC3-traps. After overnight incubation, input, flow-through (FT) and bound (B) fractions were analyzed by Western blot with an anti LC3C antibody. Lysates of A172 cells treated with Chloroquine and recombinant purified LC3C protein were used as positive controls.


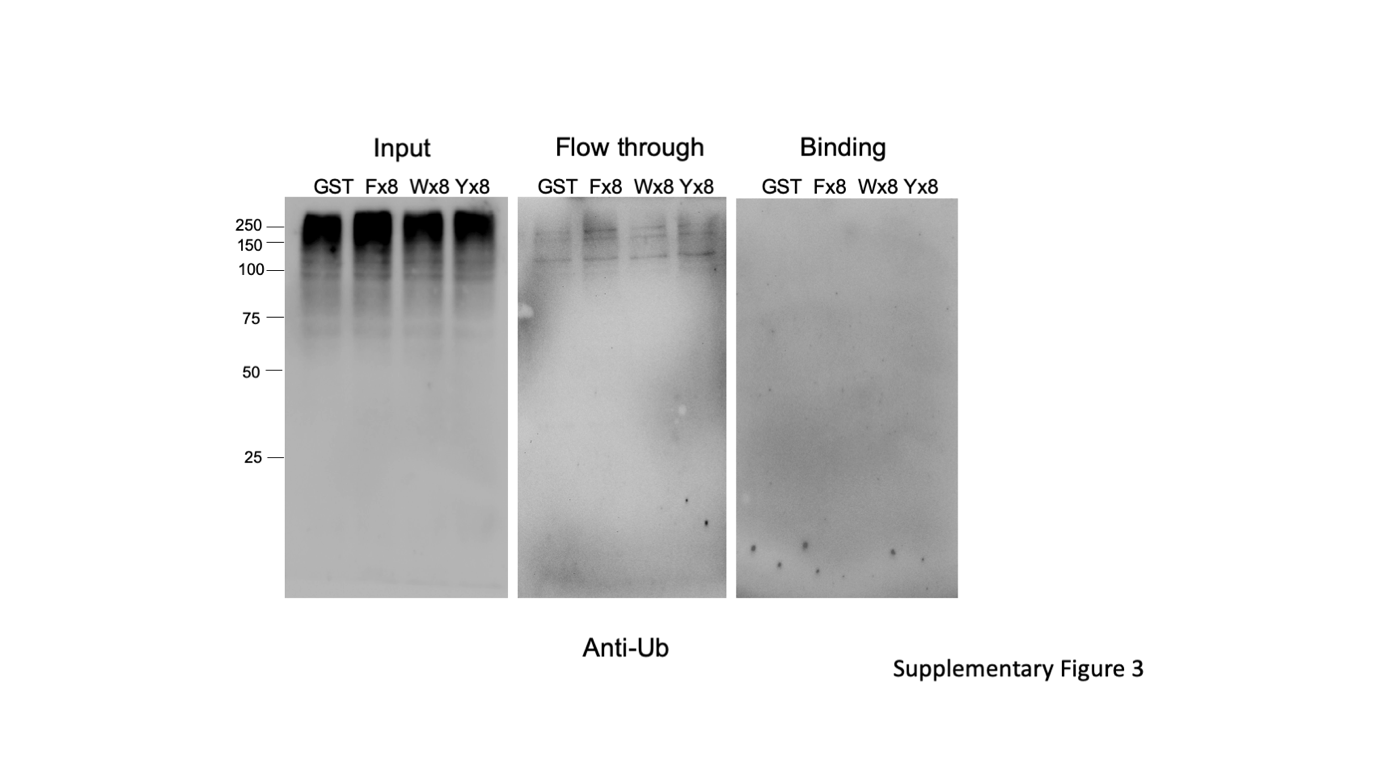


**Supplementary Figure 5. Ubiquitin chains or monomers are not captured by LC3-traps.** Cell extracts from ZBR cells treated with Bafilomycin A were used to capture ubiquitin with the indicated LC3-traps. After overnight incubation, input, flow-through (FT) and bound (B) fractions were analyzed by Western blot with the ubiquitin antibody P4D1.
